# Differential toxicity and localization of arginine-rich *C9ORF72* dipeptide repeat proteins depend on de-clustering of positive charges

**DOI:** 10.1016/j.isci.2023.106957

**Published:** 2023-05-25

**Authors:** Tamami Miyagi, Koji Ueda, Masahiro Sugimoto, Takuya Yagi, Daisuke Ito, Rio Yamazaki, Satoshi Narumi, Yuhei Hayamizu, Hiroshi Uji-i, Masahiko Kuroda, Kohsuke Kanekura

**Affiliations:** 1 Department of Molecular Pathology, Tokyo Medical University, 6-1-1 Shinjuku, Shinjuku-ku, Tokyo 160-8402, Japan; 2 Department of Pharmacology, Tokyo Medical University, 6-1-1 Shinjuku, Shinjuku-ku, Tokyo 160-8402, Japan; 3 Cancer Proteomics Group, Cancer Precision Medicine Center, Japanese Foundation for Cancer Research, 3-8-31 Ariake, Koto-ku, Tokyo 135-8550, Japan; 4 Research and Development Center for Minimally Invasive Therapies, Tokyo Medical University, 6-1-1 Shinjuku, Shinjuku-ku, Tokyo 160-8402, Japan; 5 Institute for Advanced Biosciences, KEIO University, 246-2 Mizukami, Kakuganji, Tsuruoka, Yamagata 997-0052, Japan; 6 Department of Neurology, KEIO University School of Medicine, 35 Shinanomachi, Shinjuku-ku, Tokyo 160-8582, Japan; 7 Department of Physiology, KEIO University School of Medicine, 35 Shinanomachi, Shinjuku-ku, Tokyo 160-8582, Japan; 8 Department of Molecular Endocrinology, National Research Institute for Child Health and Development, 2-10-1 Okura, Setagaya-ku, Tokyo 157-8535, Japan; 9 Department of Materials Science and Engineering, School of Materials and Chemical Technology, Tokyo Institute of Technology, 2-12-1, Ookayama, Meguro-ku, Tokyo 152-8550, Japan; 10 Department of Nanomaterials and Nanoscopy, Research Institute for Electronic Science, Hokkaido University, Kita 10 Nishi 20, North Ward, Sapporo, Hokkaido 001-0020, Japan; 11 Department of Chemistry, KU Leuven Celestijnenlaan 200F, Heverlee, 3001 Leuven, Belgium

**Keywords:** Molecular biology, Neuroscience

## Abstract

Arginine-rich dipeptide repeat proteins (R-DPRs), poly(PR) and poly(GR), translated from the hexanucleotide repeat expansion in the amyotrophic lateral sclerosis (ALS)-causative *C9ORF72* gene, contribute significantly to pathogenesis of ALS. Although both R-DPRs share many similarities, there are critical differences in their subcellular localization, phase separation, and toxicity mechanisms. We analyzed localization, protein-protein interactions, and phase separation of R-DPR variants and found that sufficient segregation of arginine charges is necessary for nucleolar distribution. Proline not only efficiently separated the charges, but also allowed for weak, but highly multivalent binding. In contrast, because of its high flexibility, glycine cannot fully separate the charges, and poly(GR) behaves similarly to the contiguous arginines, being trapped in the cytoplasm. We conclude that the amino acid that spaces the arginine charges determines the strength and multivalency of the binding, leading to differences in localization and toxicity mechanisms.

## Highlights

• Proper segregation of Arg charges is necessary for nucleolar distribution of R-DPRs
• Gly cannot fully separate Arg charges, and poly(GR) is trapped in the cytoplasm
• The structural rigidity of Pro contributes to weak but highly multivalent binding
• Poly(GR) binds to molecules in a strong, but less multivalent manner than poly(PR)

## Graphical abstract

**Figure:**
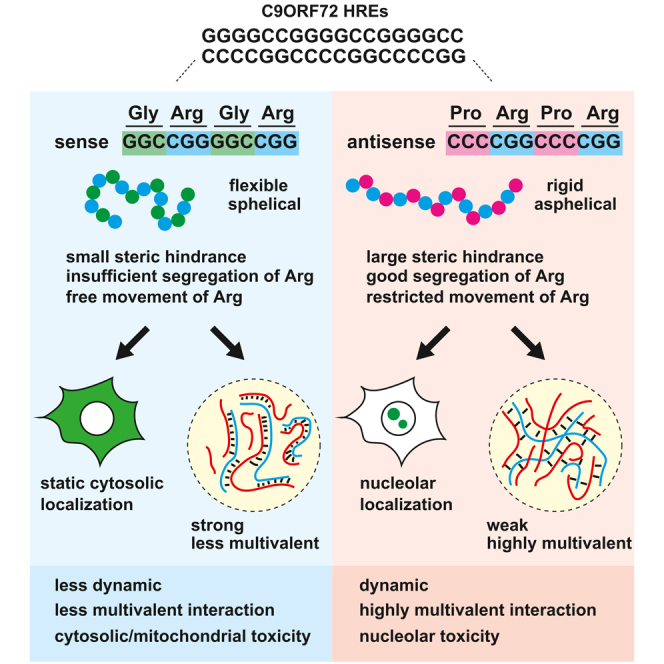

## Introduction

Amyotrophic lateral sclerosis (ALS) is a devastating neurodegenerative disease that causes progressive degeneration of upper and lower motor neurons.^1^ The most frequently mutated familial ALS-causative gene is *C9ORF72*.^2^^,^^3^ All *C9ORF72*-related ALS (C9-ALS) patients have aberrant GGGGCC hexanucleotide repeat expansions (HRE) in intron 1 of the gene. Although the precise pathophysiology of C9*-*ALS remains unclear, 1) loss of function of C9ORF72 protein,^4^ 2) HRE of DNA/RNA-mediated toxicity,^5^^,^^6^ and 3) toxic repeat-associated non-ATG translation (RAN-T) products^7^^,^^8^ have been suggested as possible causes of neurotoxicity. Among the RAN-T products, in particular, arginine-rich DPRs (R-DPRs), poly(proline-arginine: PR) and poly(glycine-arginine: GR), are reportedly highly toxic in cell and animal models.^9^^,^^10^^,^^11^

Poly(PR) and poly(GR) have similar biochemical properties in many respects. They are positively charged because of alternating arginines and have repetitive structures.^12^ These repetitively charged sequences are capable of multivalent binding (arginine works as a sticker amino acid and glycine or proline function as spacer amino acids) to macromolecules such as proteins and nucleic acids; thus, they have a strong propensity for liquid-liquid phase separation (LLPS).^13^ Indeed, both poly(PR) and poly(GR) reportedly phase-separate with various intracellular molecules^14^^,^^15^ and associate with membrane-less organelles (MLOs), disturbing their phase separation homeostasis and functions.^12^^,^^13^^,^^16^ However, there are significant differences between poly(PR) and poly(GR). In various ALS models, poly(PR) is more toxic than poly(GR).^11^^,^^12^^,^^17^ In addition, poly(PR) localizes mainly to the nucleolus, whereas poly(GR) is present in the cytoplasm and nucleolus and is often observed only in the cytoplasm,^18^^,^^19^^,^^20^^,^^21^ resulting in different interactomes.^22^^,^^23^^,^^24^ The difference in localization is confirmed for both short 20-repeat DPRs^19^ and DPRs with more than 1000 repeats, which is a physiologically-relevant size.^25^ However, the mechanism by which poly(PR) and poly(GR) localize differently, even when the repeat length is the same, remains unknown. Furthermore, it has been reported that poly(GR), but not poly(PR), exerts unique toxicity via impaired nuclear transport of TAR DNA-binding protein-43kDa (TDP-43)^26^ and disturbance of mitochondrial functions,^27^^,^^28^ but how the spacer amino acid influences protein localization, phase separation, protein-protein interactions, and toxicity remains unknown.

Here, we examined a variety of R-DPRs of different lengths, proportions, and charge distributions and found that segregation of charged arginine side chains by proline is a critical factor in promoting nucleolar incorporation of poly(PR). In addition, proline allowed interactions with acidic molecules in a highly multivalent manner *in vitro* and in the nucleolus. Still, glycine was incapable of separating the arginine charges well; hence, poly(GR) behaves like polyR, showing static localization in the cytoplasm. Poly(GR) binds to molecules in a strong, but less multivalent manner than poly(PR). These data reveal critical factors that determine biochemical differences in the R-DPRs of *C9ORF72*.

## Results

### Repeated sequences containing alternating R act as MLO-targeting signals

**Figure 1:**
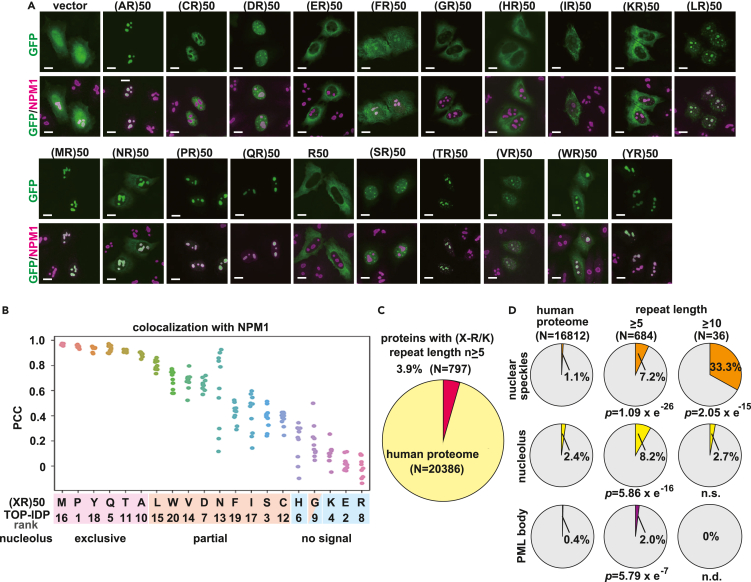
R-rich dipeptide repeat sequences function as MLO-targeting signals (A) Representative images of HeLa cells expressing indicated GFP-(XR)_50_ (X: any amino acid). The nucleolus was visualized by CoraLite555-conjugated anti-NPM1 antibody. Scale bar: 10 μm. (B) The Pearson correlation coefficient (PCC) of GFP-(XR)_50_ and NPM1. TOP-IDP is used as the disorder propensity scale. Ten cells/each were evaluated using the ImageJ EzColocalization Plugin. (C) Pie chart depicting proteins containing 5 or more repeats of (X-R/K) in the human proteome. (D) Overrepresentation of proteins containing 5 or longer repeats (middle), or proteins containing 10 or longer repeats (right) of (X-R/K) in MLO proteomes. Hypergeometric p values are indicated. n.s.: not significant. n.d.: not determined.

Poly(PR) and poly(GR) reportedly accumulate in MLOs and affect their dynamics.^12^ We observed that (PR)_50_ is exclusively localized whereas (GR)_50_ is partially localized in the nucleolus, identified by nucleolar marker nucleophosmin (NPM1) (Figure 1A). To understand the mechanism underlying their different localization, we synthesized GFP-(XR)_50_ (X: any amino acid) constructs and investigated their localization (Figure 1A). As with GFP-(PR)_50_, most GFP-(XR)_50_ proteins localized to the nucleolus, with some exceptions. Of these, (DR)_50_ and (SR)_50_ have recently been reported to act as nuclear speckle-targeting sequences (Figure S1A), whereas (ER)_50_, although its charge is neutral, like that of (DR)_50_, by reason of an unidentified mechanism, does not localize to nuclear speckles.^29^^,^^30^ Proline is the most disorder-promoting amino acid, according to the TOP intrinsically disordered protein (TOP-IDP) scale, which measures the propensity for intrinsic disorder.^31^ Thus, we hypothesized that alternate insertion of disorder-promoting amino acids among consecutive arginines would enhance nucleolar incorporation. However, there was no clear relationship between the disorder-promoting activity of amino acids inserted and nucleolar localization (Figures 1B and S1B). Among the GFP-(XRs)_50_, which have good Pearson correlation coefficient (PCC) scores with NPM1, especially (MR)_50_, (PR)_50_, (YR)_50_, (QR)_50_, (TR)_50_ and (AR)_50_ show exclusive distribution to the nucleolus (Figure 1B). We also tested the localization of (GR)_50_, (PR)_50_, (QR)_50_, R_50_ and (YR)_50_ in a motor neuronal cell line, NSC34 cells, and obtained consistent results (Figure S1C).

We next investigated whether (XR) repeats present in natural protein function as MLO-targeting sequences. Because sequences with repeating K also migrate to MLOs,^29^^,^^32^ we decided to count R and K equally here. We searched for (X-R/K) repeats in the human proteome in the UniProt database.^33^ We found 797 proteins with 5 or more repeats of (X-R/K) among 20,386 proteins in the human proteome (Figure 1C), and 40 of these have 10 or more repeats. We next examined amino acid occurrences in the repeats. Proteins with ≥5 repeats tend to contain D/E/S, which was further enhanced in sequences containing more than 10 repeats (Figure S1D). D and E are acidic amino acids, and S acts like an acidic amino acid when phosphorylated by endogenous serine kinases.^29^ To determine whether these (X-R/K) repeats contribute to the distribution to MLOs, we investigated the subcellular localization of repeat-containing proteins. In the human proteome, localizations of 16,812 proteins are available in UniProt. The proteins localized to nuclear speckles occupying 1.1% of the human proteome, but the overrepresentation of nuclear speckle proteins is confirmed in the proteome containing 5 or more (X-R/K) repeats (7.2%, hypergeometric p value = 1.09×e^−26^), especially in the proteome with 10 or more repeats (33.3%, hypergeometric p value = 2.05×e^−15^) (Figure 1D). Similarly, nucleolar proteins (8.2%, hypergeometric p value = 5.86×e^−16^) and proteins in promyelocytic leukemia (PML) body (2.0%, hypergeometric p value = 5.79×e^−7^) are overrepresented in the proteome with 5 or more repeats. These results indicate that the appearance of periodic basic amino acids contributes to localization to the MLO.

Next, we investigated whether alternate positioning of arginines is important for localization of the protein to the nucleolus or whether the order is irrelevant, as long as it is positively charged. When we expressed a (PR)_50_ variant (P_16_R_16_)_3_ with the same net charge, but different charge distribution [two more arginines were added to its C-terminus to equalize the number of arginines (N = 50)], it did not localize to the nucleolus, but to the cytosol (Figures S2A and S2B).^34^ To investigate whether this phenomenon is specific to (PR)_50_ or universal, we examined the effect of charge distribution on nucleolar localization of (QR)_50_, (YR)_50_, and (GR)_50_. As with GFP-(P_16_R_16_)_3_, uneven charge distributions with the same net charge resulted in loss of nucleolar localization, and GFP-(Q_16_R_16_)_3_, GFP-(Y_16_R_16_)_3_, and GFP-(G_16_R_16_)_3_ showed cytosolic distribution (Figure S2C). Although consecutive basic amino acids act as nucleolar targeting signals,^32^ GFP-(X_16_R_16_)_3_ paradoxically localized to the cytoplasm. Next, we examined whether lysine, which also has a positive charge, also exhibits similar effects. We tested (PK)_50_ variants, and as with arginine, cytoplasmic localization was enhanced by uneven distribution of lysine, but unlike arginine, GFP-(P_16_K_16_)_3_ remained partially localized to the nucleolus (Figure S2D). We tested whether this rule could be applied to natural proteins. The US11 protein of herpes simplex virus (HSV) type 1 has 24 (X-P-R) repeats and localizes to the nucleolus,^35^ but uneven distribution of arginines resulted in the loss of localization to the nucleolus and the US11 mutant localized to the cytoplasm (Figure S2E). Human ribosomal protein L29 (RPL29), which has repetitive basic amino acids, localizes to the nucleolus and cytoplasm.^36^ Still, by changing the distribution of basic amino acids without changing the net charge, it lost nucleolar incorporation and localized to the cytoplasm (Figure S2F). Furthermore, when lysines of wild-type RPL29 were substituted for arginines, its targeting to the nucleolus was enhanced and when arginines of wild-type RPL29 were substituted for lysines, its targeting to the nucleolus was weakened, indicating that arginine has a greater impact on nucleolar localization than lysine (Figure S2F). These data indicate that periodic appearance, rather than contiguous arginines is important for nucleolar distribution.

### Poly(GR) has characteristics similar to those of consecutive R, and localizes to the cytosol

In *C9ORF72*-ALS, both poly(PR) and poly(GR) are toxic *in vitro* and *in vivo.*^9^^,^^10^^,^^16^^,^^37^ Both poly(PR) and poly(GR) contain 50% arginine, and the positive charge of arginine allows them to interact with acidic macromolecules, including nucleic acids and proteins, resulting in neurotoxicity. However, poly(PR) and poly(GR) reportedly activate different neurotoxic pathways, even though they contain the same ratio of arginine.^27^^,^^38^ One possible explanation for this is the difference in their subcellular localization.

The principles of protein targeting to the nucleolus are well-studied and this targeting is mediated by a charge-dependent mechanism.^39^ Six consecutive arginines are sufficient for the peptide to distribute to the nucleolus, and an isoelectric point above 12.6 is sufficient for targeting to the nucleolus.^32^ The web-based nucleolar localizing signal (NoLS) detector (NoD), which predicts the presence of NoLS in proteins, also reveals that the positive charge rather than a specific motif is responsible for nucleolar localization.^40^ According to the NoD, both (PR)_50_ and (P_16_R_16_)_3_ are recognized as NoLS (Figure S3A). However, GFP-(PR)_50_ localized to the nucleolus, whereas GFP-(P_16_R_16_)_3_ localized to the cytosol (Figure S2B). In addition, GFP-(GR)_50_ localized to both the nucleolus and cytosol despite the same isoelectric point as GFP-(PR)_50_,^19^ indicating that a factor other than net charge determines nucleolar localization of the protein.

**Figure 2:**
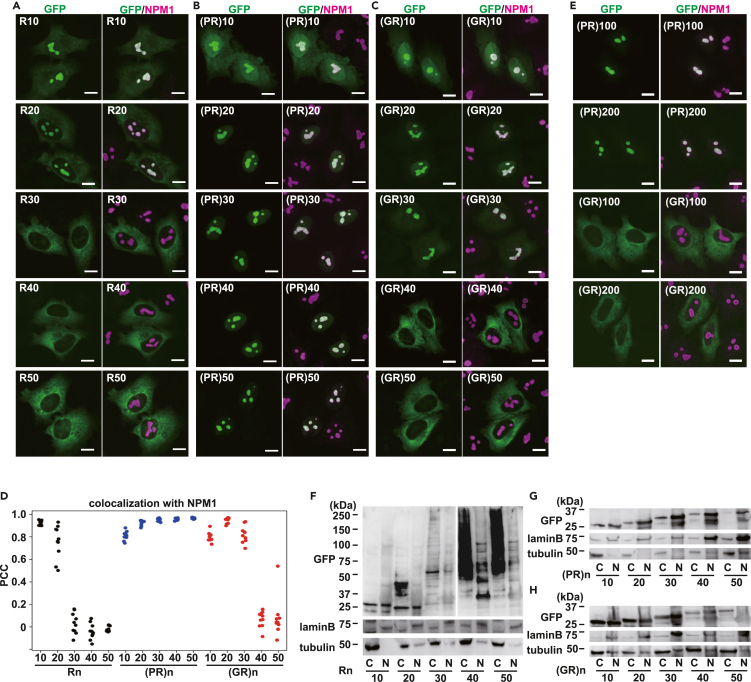
PolyR and poly(GR), but not poly(PR), have a length threshold for nucleolar localization (A) Representative images of HeLa cells expressing different repeat lengths of GFP-polyR. The nucleolus was visualized by CoraLite555-conjugated anti-NPM1 antibody. Scale bar: 10 μm. (B) Representative images of HeLa cells expressing different repeat lengths of GFP-poly(PR). Scale bar: 10 μm. (C) Representative images of HeLa cells expressing different repeat lengths of GFP-poly(GR). Scale bar: 10 μm. (D) The PCC of GFP-polyR, GFP-poly(PR) or GFP-poly(GR) and NPM1. Ten cells/each were evaluated. (E) Representative images of HeLa cells expressing GFP-(PR)_100_, GFP-(PR)_200_, GFP-(GR)_100_ or GFP-(GR)_200_, respectively. Scale bar: 10 μm. (F–H) Immunoblot of cytosolic fractions (C) and nuclear fractions (N) of HeLa cells expressing different repeat lengths of GFP-polyR (F), GFP-poly(PR) (G), or GFP-poly(GR) (H). Theoretical molecular weights of GFP-R_50_, GFP-(PR)_50_ or GFP-(GR)_50_ are 34.8 kDa, 37.6 kDa, 39.6 kDa, respectively. Lamin B is a nuclear marker and tubulin is a cytosol marker, respectively.

To clarify the mechanism, we first investigated the effect of the number of arginine residues on nucleolar localization. Arginine, a positively charged amino acid with a dipole moment, is responsible for the protein-protein interaction of poly(PR).^15^ When we overexpressed R_10_ and R_20_ in HeLa cells, they localized to the nucleolus, as reported previously.^32^^,^^39^ However, although consecutive R is recognized as an NoLS by the NoD webserver, constructs of R_30_ or longer localized to the cytosol, but not to the nucleolus (Figure 2A). In contrast, when we overexpressed poly(PR), it localized to the nucleolus, and the longer it was, the more exclusively the localization became (Figure 2B). Next, we tested the poly(GR) with different repeat lengths. When the repeat length of poly(GR) was increased, consistent localization to the nucleolus was observed until a repeat number of 30 (Figure 2C). However, as the length of poly(GR) was further increased, localization to the nucleolus peaked at a certain point and then shifted to cytoplasmic localization, which is very similar to the behavior of contiguous R (Figure 2A). The PCC of the GFP signal with a nucleolar marker NPM1 signal indicated that the PCC decreased as the lengths of consecutive R and poly(GR) increased, but for poly(PR), the PCC with the NPM1 signal increased for longer poly(PR) repeat lengths (Figure 2D). We further tested longer constructs of poly(PR) and poly(GR). Because the HREs longer than 200 are considered as very likely pathogenic,^41^ we synthesized (PR)_100_, (PR)_200_, (GR)_100_ and (GR)_200_ and examined their localization. Consistent with (PR)_50_ and (GR)_50_, (PR)_100_ and (PR)_200_ exclusively localized to the nucleolus whereas (GR)_100_ and (GR)_200_ localized to cytosol (Figure 2E). Supporting these data, poly(PR) of physiologically relevant size, (PR)_1100_, still exclusively localizes to the nucleolus.^25^

To biochemically validate these results, fractionation analyses were performed. PolyR produced smears whose molecular weights were larger than expected in the cytoplasmic fraction. Poly(PR) and poly(GR) showed bands in the expected sizes. Poly(PR) enhanced the nuclear signal as its length increased, whereas poly(GR) shifted its localization from the nucleus to the cytoplasm when its length increased (Figures 2F–2H). The effect of repeat length on nucleolar localization was also tested for poly(YR) and poly(QR), which migrated to the nucleolus similarly to poly(PR) (Figure 1A). Poly(QR) localized to the nucleolus more efficiently when the repeat length was increased, as with poly(PR) (Figure S3B). (YR)_10_ did not localize to the nucleolus, but to the nucleoplasm, but (YR)_30_ and (YR)_50_ efficiently localized to the nucleolus (Figures S3C and S3D). This suggests that in general, poly(XR) more efficiently localizes to the nucleolus when the repeat length is increased and that poly(GR) is exceptional in this respect. As reported previously, consecutive lysines also act as a nucleolar migration signal.^32^ Therefore, we generated polyK of different lengths as well, and tested their subcellular localization (Figures S3E and S3F). We found that co-localization of polyK with NPM1 decreased as the length increased, as was the case with polyR, but a faint signal in the nucleolus remained even at a repeat length of 50 (Figures S3E and S3F).

Nuclear import of proteins is mediated by both passive diffusion and active transport via importin family proteins.^42^ Importin family proteins bind to the nuclear localizing signal (NLS) and the importin complexes move through the nuclear pore. To test whether loss of nucleolar distribution of (GR)_50_ could be reverted by addition of NLS, we fused three tandem repeats of NLS of SV40 large T antigen (PKKKRKVD)^43^ to the N-terminus of GFP-(GR)_50_ and tested its localization, but the NLS did not affect localization of (GR)_50_ (Figures S4A and S4B). We did not observe any effect of the NLS on R_50_ localization either. We next investigated whether increased cytosolic distribution of R_50_ and (GR)_50_ is static or a result of an exaggerated nucleocytoplasmic export system. To test this, we treated cells with a Chromosomal maintenance 1 (CRM1, also known as exportin-1) inhibitor, Leptomycin B (LMB).^44^ The shuttle-tdTomato (s-tdTomato), which carries both the NLS and nuclear exporting signal (NES) and which works as a fluorescent reporter of nucleocytoplasmic transport,^45^ was localized in both the cytoplasm and nucleus in untreated cells, whereas in LMB-treated cells, nucleocytoplasmic export was arrested and s-tdTomato accumulated in the nucleus (Figure S4C). However, localization of R_50_ and (GR)_50_ to the cytoplasm was not affected by LMB, indicating that the distribution of R_50_ and (GR)_50_ to the cytoplasm was static rather than enhanced CRM1-mediated nucleocytoplasmic export. Arginine has a strong positive charge and promotes intermolecular interactions. Contiguous arginines might align well with acidic sequences nearby,^46^ suggesting that polyR is strongly bound to cytosolic molecules, as shown by the smear in the fractionation experiment (Figure 2F). To evaluate the macromolecular environment around R_50_, we fused CRONOS (crowding sensor with mNeonGreen and mScarlet-I), a Förster resonance energy transfer (FRET)-based macromolecular crowding sensor, with R_50_ (Figure S4D).^47^ CRONOS consists of mNeonGreen and mScarlet-I connected by a flexible linker, similar to the mCerulean-mCitrine-based sensor as previously reported,^48^ and once the macromolecular crowding level increases, the distance between these two fluorescent proteins diminishes, resulting in higher FRET efficiency. The increased FRET efficiency of the CRONOS reporter suggested that GFP-R_50_ is more likely to have enhanced macromolecular crowding because of interactions with surrounding molecules (Figures S4E and S4F). These results suggest that (GR)_50_ and R_50_ do not diffuse freely in the cytoplasm, but rather bind strongly to intracytoplasmic molecules, making them difficult to translocate to nucleus.

### Poly(GR) binds strongly to surrounding molecules

**Figure 3:**
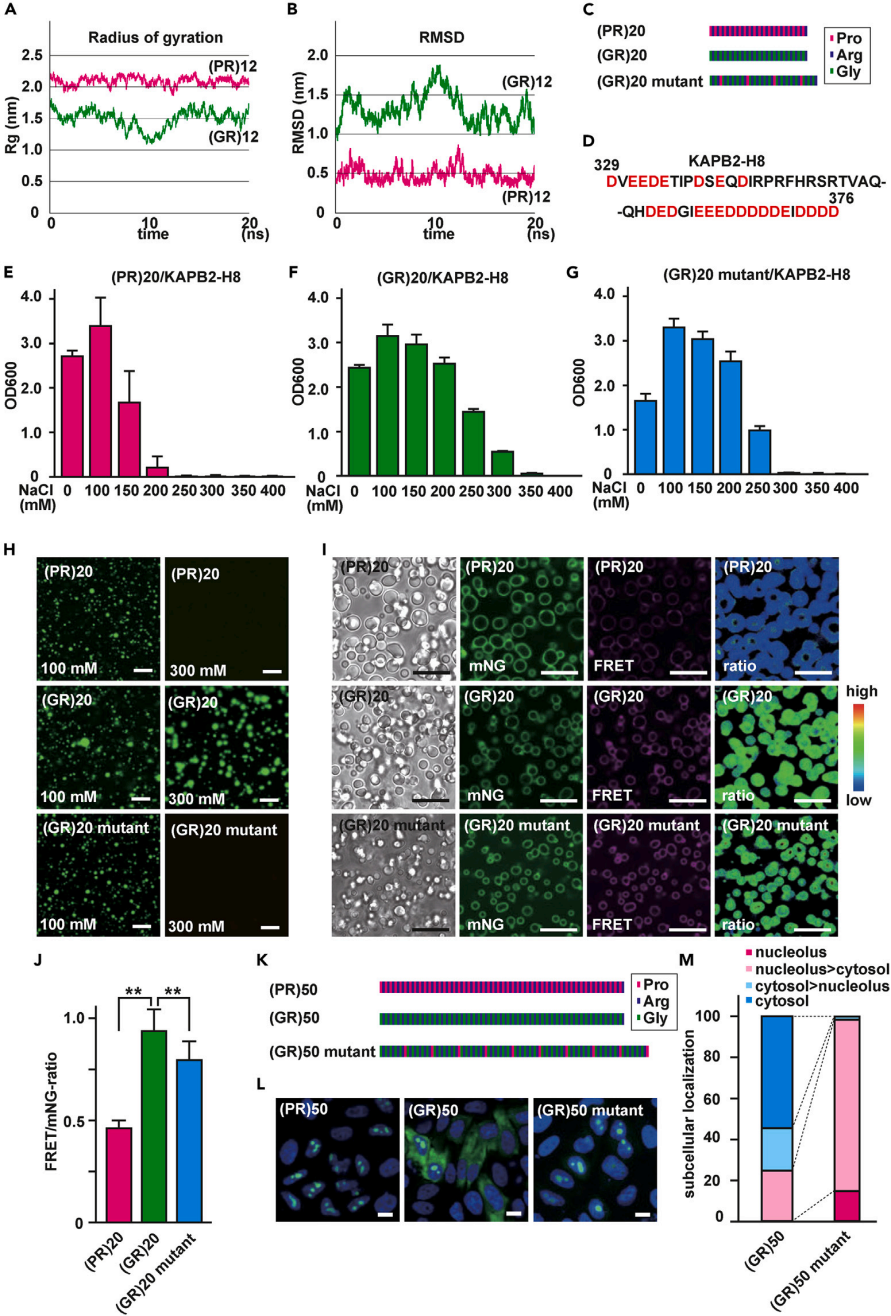
Poly(GR) binds to acidic biomolecules more strongly than poly(PR) (A) Radius of gyration (Rg) of (PR)_12_ and (GR)_12_ obtained from MD simulation. (B) Root-mean-square deviation (RMSD) of (PR)_12_ and (GR)_12_ obtained from MD simulation. (C) Diagram of amino acid distributions in (PR)_20_, (GR)_20_ and (GR)_20_ mutant. (D) The amino acid sequence of KAPB2-H8. Acidic amino acids are highlighted in red. (E) Turbidity of solutions containing (PR)_20_ and KAPB2-H8 with different salt concentrations. N = 3. Error bars show ±S.D. (F) Turbidity of solutions containing (GR)_20_ and KAPB2-H8 with different salt concentrations. N = 3. Error bars show ±S.D. (G) Turbidity of solutions containing (GR)_20_ mutant and KAPB2-H8 with different salt concentrations. N = 3. Error bars show ±S.D. (H) Confocal images of mixed solutions of KAPB2-H8 in combination with (PR)_20_, (GR)_20_ or (GR)_20_ mutant in buffer containing 100 mM NaCl or 300 mM NaCl. Droplets were visualized by addition of 100 nM of FITC-labeled (PR)_20_, FITC-labeled (GR)_20_ or FITC-labeled (GR)_20_ mutant, respectively. Scale bar: 10 μm. (I) Confocal images of mixed solutions of KAPB2-H8 in combination with (PR)_20_, (GR)_20_ or (GR)_20_ mutant in buffer containing 1 μM of CRONOS-KAPB2-H8. Ratiometric images of FRET/mNG are pseudocolored. Scale bar: 10 μm. (J) FRET/mNG ratios of droplets of (PR)_20_, (GR)_20_ or (GR)_20_ mutant (N120 droplets/each). Error bars show ±S.D. ∗∗: p < 0.01. (K) Diagram of amino acid distributions in (PR)_50_, (GR)_50_ and (GR)_50_ mutant. (L) Representative images of HeLa cells expressing GFP-(PR)_50_, GFP-(GR)_50_ or GFP-(GR)_50_ mutant. Nuclei were visualized with DAPI. Scale bar: 10 μm. (M) Stacked column chart indicating the subcellular localization of GFP-(GR)_50_ (N = 126 cells) or GFP-(GR)_50_ mutant (N = 123 cells).

Glycine is the smallest amino acid, and because of its side chain of a single hydrogen atom, glycine confers flexibility on peptide structure. In contrast, proline has a pyrrolidine side chain that restricts peptide flexibility, contributing to structural rigidity. To dissect biochemical differences between poly(PR) and poly(GR), we performed a molecular dynamics (MD) simulation of (PR)_12_ and (GR)_12_ using GROMACS, to estimate their folding status.^49^ The radius of gyration (Rg) of (PR)_12_ is larger than that of (GR)_12_, indicating that (GR)_12_ can fold more compactly than (PR)_12_ (Figure 3A). The larger root-mean-square deviation (RMSD) of (GR)_12_ was consistent with the expected higher flexibility of (GR)_12_ (Figure 3B). These results were consistent with former MD simulation results of poly(PR) and poly(GR).^50^ Previous computational and experimental studies revealed that the distribution of charged residues affects electrostatic forces, and consecutive charged residues have especially strong charge correlations because of the sequence alignment of two nearby chains, which is advantageous for strong binding.^34^^,^^46^ The highly flexible nature of poly(GR) may be beneficial for sequence alignment of nearby molecules, similar to consecutive arginines. If this is true, poly(GR) binding to surrounding acidic molecules is stronger than that of poly(PR).

To test this hypothesis, we first evaluated fluidity of molecules phase-separated with poly(GR) or poly(PR) by fluorescence recovery after photo bleaching (FRAP) analysis. When mixed with poly-rA RNA containing tetramethylrhodamine (TAMRA)-labeled rA_15_, all R_12_, (GR)_12_, and (PR)_12_ underwent LLPS (Figure S5A). The FRAP analysis showed that fluidity of (PR)_12_ droplets was higher than that of (GR)_12_ or R_12_ (Figure S5B), indicating that R_12_ and (GR)_12_ bound more tightly to RNA than did (PR)_12_. It is possible that efficient localization to the nucleolus can be explained by strong affinity for nucleolar molecules. To compare the affinity of GFP-R_20_, GFP-(PR)_20_ and GFP-(GR)_20_ to the nucleolus in live cells, we performed FRAP analysis in the nucleolus (Figure S5C). GFP-(PR)_20_ loosely interacted with nucleolar molecules and maintained high fluidity, while GFP-R_20_ and GFP-(GR)_20_ strongly interacted with nucleolar molecules and had low fluidity, which is consistent with the *in vitro* FRAP analysis (Figure S5B). This finding was further substantiated by FRAP analysis of nucleolar GFP-(GR)_50_ and GFP-(PR)_50_ (Figure S5D). To directly compare binding strength between poly(PR) or poly(GR) and acidic molecules, we measured critical salt concentration, which is the minimal concentration of NaCl needed to dissolve the phase-separated droplets. To this end, we used (PR)_20_, (GR)_20_, and (GR)_20_ mutants with insertion of proline in every (GR)_5_ [(GR)_20_ mutant] (Figure 3C), and the acidic H8 loop of karyopherin β2 (KAPB2, also known as transportin-1) (KAPB2-H8) (Figure 3D). The interaction between R-DPRs and KAPB2-H8 was confirmed by nuclear magnetic resonance (NMR).^26^^,^^51^^,^^52^ Direct binding of poly(GR) to KAPB2 reportedly inhibits nuclear translocation of TDP-43.^26^ All (PR)_20_, (GR)_20_ and (GR)_20_ mutant underwent LLPS with the KAPB2-H8 peptide in buffer containing 100 mM NaCl. When we increased the concentration of NaCl, (PR)_20_ droplets disappeared at 250 mM NaCl whereas (GR)_20_ droplets remained until 300 mM NaCl (Figures 3E and 3F), indicating that (GR)_20_ bound more tightly to KAPB2-H8 than did (PR)_20_. Importantly, (GR)_20_ mutant droplets disappeared at 300 mM NaCl even though they carried 20 (GR) repeats, as in (GR)_20_ (Figures 3G and 3H). Because (PR)_20_ showed higher fluidity in LLPS droplets, we speculated that the inside of (PR)_20_ droplets might be less crowded. To examine whether proline residues actually render droplets more fluid, we measured macromolecular crowding levels in phase-separated droplets formed by KAPB2-H8 and (PR)_20_, (GR)_20_ or (GR)_20_ mutant. In this experiment, we added recombinant CRONOS C-terminally fused with KAPB2-H8 peptide to LLPS droplets. The CRONOS-KAPB2-H8 reported macromolecular crowding in the droplets, and the (GR)_20_ droplets showed the highest macromolecular crowding (Figures 3I and 3J). In contrast, periodic insertion of proline into every 5 (GR) repeats attenuated the crowding, and (PR)_20_ droplets showed the lowest degree of macromolecular crowding, indicating that proline residues indeed contributed to the formation of loosely packed droplets. Next, we tested the effect of periodic insertion of proline every five (GR) repeats on localization of (GR)_50_ (Figure 3K). Localization of (GR)_50_ to the nucleolus was drastically enhanced by insertion of prolines (Figures 3L, 3M, and S5E). FRAP analysis of (GR)_50_ mutant showed slightly better recovery (Figure S5F), indicating that (GR)_50_ can distribute to the nucleolus if intermolecular interactions are attenuated.

### Alternate insertion of Pro, but not other amino acids, promotes multivalent interactions

**Figure 4:**
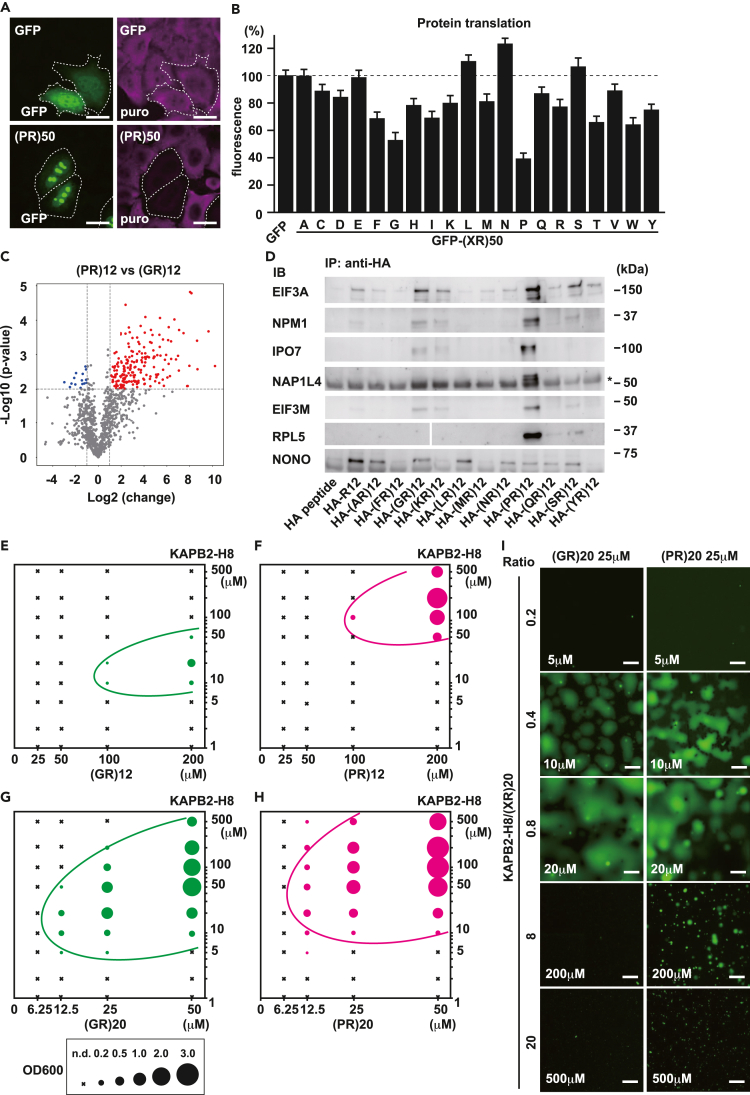
Alternate proline residues enhance multivalent interactions with acidic molecules (A) Immunofluorescence of newly synthesized proteins labeled with puromycin (magenta) in HeLa cells expressing GFP (green) or GFP-(PR)_50_. Scale bar: 10 μm. (B) Fluorescence intensities of newly synthesized proteins labeled with puromycin in HeLa cells expressing GFP-(XR)_50_. N = 60 cells/each. Error bars show ±S.D. (C) A volcano plot indicating differences between interactomes of HA-(PR)_12_ and HA-(GR)_12_. Interactome data were obtained from a previous study.^34^ (D) IP-IB comparing the degree of interactions of HA-(XR)_12_ with indicated proteins. The asterisk shows the signal of the IgG heavy chain. (E) Phase diagram of (GR)_12_ of indicated concentrations mixed with different concentrations of KAPB2-H8. The area of the filled circle indicates the value of OD600. n.d.: not detected. (F) Phase diagram of (PR)_12_ of indicated concentrations mixed with different concentrations of KAPB2-H8. (G) Phase diagram of (GR)_20_ of indicated concentrations mixed with different concentrations of KAPB2-H8. (H) Phase diagram of (PR)_20_ of indicated concentrations mixed with different concentrations of KAPB2-H8. (I) Representative images of the phase separated droplets consisting of 25 μM of each R-DPR and different concentrations of KAPB2-H8. Droplets were visualized by addition of 100 nM of FITC-labeled (GR)_20_ or FITC-labeled (PR)_20_, respectively. Scale bar: 10 μm.

As reported previously, poly(PR) and poly(GR) inhibit protein translation *in vitro* and *in vivo.*^12^^,^^16^^,^^53^^,^^54^ To test whether this inhibitory effect is specific to poly(PR) and poly(GR) or is a universal feature of poly(XR), we performed a puromycin-based SUnSET assay to monitor protein translation in cells^55^ (Figure 4A). Most GFP-(XR)_50_ exerted mild suppression, whereas GFP-(PR)_50_ showed the most potent inhibitory effect on protein translation (Figure 4B). To dissect the mechanism underlying the strong toxicity of poly(PR), we investigated the nature of protein-protein interactions of poly(PR). When proline is alternately inserted into consecutive arginine sequences, it weakens the intermolecular interaction, but promotes multivalent interactions, resulting in a stronger propensity for LLPS with acidic proteins.^34^ A comparison of the quantitative interactome analysis of R_12_, (GR)_12_, and (PR)_12_ shows that the interactome of (GR)_12_ is similar to that of R_12_,^34^ but (PR)_12_ is very different from (GR)_12_, with many signals enhanced (Figure 4C). To examine whether this signal enhancement of interacting proteins is proline-specific or whether it is also observed for other amino acids, depending on the size and hydrophobicity of their side chains, we performed immunoprecipitation (IP)-immunoblot analysis (IB) by mixing synthetic HA-(XR)_12_ peptides with cell lysates (Figure 4D). In this experiment, we chose candidate interacting proteins based on our previous study,^34^ in which we detected proteins common to the interactomes of R_12_, (GR)_12_, and (PR)_12_ and confirmed that spacer amino acids influence affinity to these proteins. Non-POU domain-containing octamer-binding protein (NONO) was chosen as a control that did not differ between R_12_ and (PR)_12_. We found that signal intensities of binding proteins were enhanced only in (PR)_12_. This enhancement was specific because some proteins such as NONO interacted similarly with all (XR)_12_ peptides (Figure 4D). This suggests that proline is particularly prone to forming high-multivalent interactions, which may account for the phenotypic difference between poly(PR) and poly(GR). To further prove this, we mixed poly(PR) or poly(GR) with KAPB2-H8 peptide in different ratios and monitored their phase separation. If poly(PR) is capable of interacting with a larger number of molecules, poly(PR) can phase separate and form droplets with a higher molar ratio of KAPB2-H8 than poly(GR). Because the length of DPR affects its propensity for phase separation, we tested peptides with different repeat numbers, 12 and 20. The phase diagram shows that (PR)_12_ phase-separated with higher molar ratio of KAPB2-H8 than (GR)_12_ (Figures 4E and 4F). This result was further emphasized with peptides of repeat length 20. 25 μM of (PR)_20_ can undergo LLPS with 500 μM of KAPB2-H8 (molar ratio = 1:20) (Figures 4G–4I). These results indicate that poly(PR) is capable of binding to a larger number of molecules per peptide than poly(GR) and forms phase-separated droplets.

### Adequate de-clustering of arginine charges is necessary for R-DPR distribution to the nucleolus

**Figure 5:**
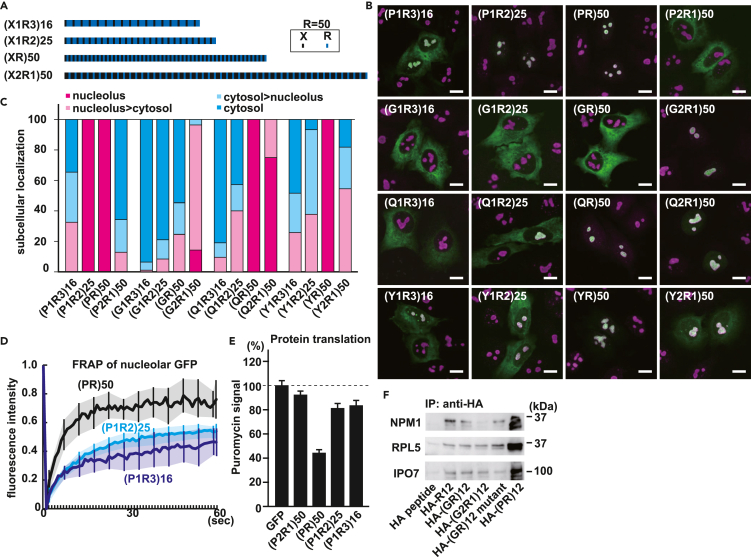
Appropriate segregation of arginine charges is essential for nucleolar incorporation (A) Diagram of ratio variants of (XR)_50_. (B) Representative images of HeLa cells expressing GFP-(XR)_50_ ratio variants. The nucleolus was visualized by CoraLite555-conjugated anti-NPM1 antibody. Scale bar: 10 μm. (C) 100% stacked column chart indicating subcellular localizations of GFP-(XR)_50_ ratio variants. N = 28–110 cells/each. (D) FRAP recovery of GFP-(PR)_50_ in the nucleolus as compared to GFP-(P_1_R_2_)_25_ and GFP-(P_1_R_3_)_16_. Mean values and standard deviations from 9 independent analyses are shown. (E) Fluorescence intensities of newly synthesized proteins labeled with puromycin in HeLa cells expressing each GFP-(PR)_50_ ratio variant. N = 60 cells/each. Error bars show ±S.D. (F) IP-IB comparing the degree of interactions of HA-R_12_, -(GR)_12_ variants or HA-(PR)_12_ with indicated proteins.

The ratio of proline (spacer) to arginine (sticker) in the poly(PR) determines the nature of the poly(PR) droplets, and the segregation of adjacent arginines by proline is critical for the toxicity of poly(PR).^34^ To assess the influence of the sticker-spacer ratio on nucleolar incorporation, we tested nucleolar localization of variants of (PR)_50_ and (GR)_50_ with different ratios of sticker and spacer (Figure 5A). We also tested the effect in (QR)_50_ and (YR)_50_ because they showed a good PCC with nucleolar NPM1 signals (Figures 1A and 1B), and glutamine and tyrosine are known as typical components of LLPS-prone proteins.^56^ Here, spacer X separates the charges of neighboring arginines, and in the case of X:R = 2:1, the distance between adjacent arginines is doubled compared to the 1:1 case. If X:R = 1:2 or 1:3, two or three consecutive arginines will appear, and one spacer will separate the consecutive charges for every two or three arginines. Because contiguous arginines above a certain threshold are trapped in the cytoplasm (Figure 2A), it is presumed that spacer amino acids, which are efficient in separating contiguous arginine charges, help escape from entrapment and facilitate entry into the nucleolus. If the distance between arginines is too great, the clustering of positive charges necessary for nucleolar distribution is lost, and accumulation in the nucleolus is limited. First, we monitored subcellular localization of (PR)_50_ variants containing 50 arginines with different P:R ratios. (P_1_R_3_)_16_ was found in both the cytoplasm and the nucleolus, whereas (P_1_R_2_)_25_ and (PR)_50_ were exclusively localized in the nucleolus (Figures 5B and 5C). (P_2_R_1_)_50_ was found in the cytosol without nucleolar distribution, indicating that segregation of arginine charges by proline is efficient (Figure 5B). In the case of glycine, (G_1_R_3_)_16_ and (G_1_R_2_)_25_ localized to the cytoplasm, whereas (GR)_50_ partially localized to the nucleolus in 22% of cells (Figure 5C). Importantly, (G_2_R_1_)_50_ showed dramatically increased localization to the nucleolus, suggesting that a single glycine was not sufficient to separate the arginine charges and that insertion of two glycines between arginine separated the charge enough to shift localization from the cytosol to the nucleolus. When (QR)_50_ and (YR)_50_ were tested the same way, insertion of two glutamines between arginines resulted in decent migration to the nucleolus, whereas insertion of two tyrosines between arginines attenuated the degree of migration to the nucleolus, suggesting that the ability to segregate the charges of consecutive arginines is P > Y > Q > G. These observations were also confirmed in the motor neuronal NSC34 cells (Figure S6). These results indicate that the difference in subcellular localization of poly(PR) and poly(GR) is because of the difference in their ability to separate the arginine charges. The FRAP analysis showed that (P_1_R_3_)_16_ is most tightly bound to nucleolar components, followed by (P_1_R_2_)_25_ and (PR)_50_, indicating that strong binding to nucleolar molecules does not necessarily mean efficient incorporation into the nucleolus (Figure 5D). Although both GFP-(P_1_R_3_)_16_ and GFP-(P_2_R_1_)_50_ localized to the cytosol, we speculated that the molecular mechanisms underlying their localization are different.

To investigate this, we overexpressed GFP-(P_1_R_3_)_16_ or GFP-(P_2_R_1_)_50_ in combination with s-tdTomato in HeLa cells and treated them with LMB. GFP-(P_1_R_3_)_16_ remained in the cytoplasm, whereas GFP-(P_2_R_1_)_50_ accumulated in the nucleus (Figures S7A and S7B), indicating that GFP-(P_1_R_3_)_16_ was statically localized in the cytoplasm, whereas GFP-(P_2_R_1_)_50_ freely moved between the nucleus and cytoplasm and was LMB-sensitive. The degree of inhibition of protein translation was also affected by the ratio of P:R and (PR)_50_ showed the strongest inhibitory effect (Figure 5E). To examine whether the greater migration rate of (G_2_R_1_)_50_ and (GR)_50_ mutant [one proline inserted into every (GR)_5_] (Figure 3K) to the nucleolus depends on mimicking the charge separating property or the high-multivalent interaction of (PR)_50_, we performed IP-IB using (G_2_R_1_)_12_ and (GR)_12_ mutant. We confirmed that neither (G_2_R_1_)_12_ nor (GR)_12_ mutant developed higher multivalent interactions (Figure 5F). Therefore, segregation of arginine charges rather than high-multivalent interactions is important for nucleolar incorporation. The inability of glycine to separate the charges of arginine probably depends on the size of side chain and the flexibility. Alanine has the next smallest side chain after glycine (Figure S8A), and LLPS droplets with (AR)_12_ showed similar biochemical characteristics to those of R_12_ and (GR)_12_, such as irregular shapes of the phase-separated droplets and the limited recovery rate of FRAP analysis (Figures S8B and S8C). However (AR)_50_ is localized almost exclusively to the nucleolus, indicating that the methyl group of the side chain of alanine, which limits rotation of the peptide main chain, has a significant effect on subcellular localization (Figure 1A).^57^

### De-clustering of arginine charges contributes to differences between the interactomes and toxicity of R-DPRs

**Figure 6:**
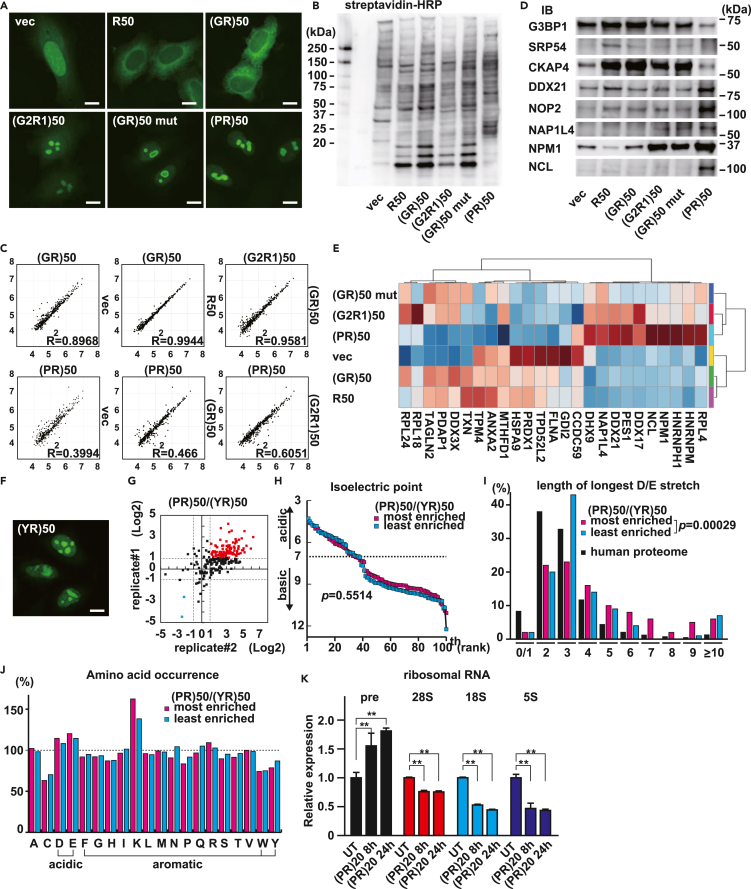
De-clustering of arginine charges contributes to differences between the interactomes of poly(GR) and poly(PR) (A) Representative images of HeLa cells expressing TurboID control vector (vec), TurboID-R_50_, TurboID-(GR)_50_, TurboID-(G_2_R_1_)_50_, TurboID-(GR)_50_ mutant, or TurboID-(PR)_50_. Biotinylated proteins were visualized with AlexaFluor488-conjugated streptavidin. Scale bar: 10 μm. (B) Proteins biotinylated with TurboID were analyzed by SDS-PAGE followed by visualization with HRP-conjugated streptavidin. (C) Scatterplots of interactome data. Each dot represents the average of triplicate experiments. The X and Y axes show signal intensities as -Log_10_ values. (D) Immunoblot analyses of biotinylated proteins pulled down by streptavidin beads with indicated antibodies. (E) A heatmap displaying averaged data of triplicate samples clustered by their Pearson correlation coefficients. Features showing high variances among the top 25 groups are visualized. The colors red-blue-white indicate higher, lower, and average. (F) Representative images of HeLa cells expressing TurboID-(YR)_50_. Biotinylated proteins were visualized with AlexaFluor488-conjugated streptavidin. Scale bar: 10 μm. (G) A scatterplot indicating enrichment of identified proteomes. Analyses were performed in duplicate, and proteins with signal intensities that were at least doubled in the interactome of (PR)_50_, compared with that of (YR)_50_ are colored in red. (H) Isoelectric points of the 100 most enriched proteins and the 100 least enriched proteins in the interactome of (PR)_50_ when compared with that of (YR)_50_. Proteins were sorted in order of pI. (I) Histogram of the longest D/E stretches in the 100 most enriched and 100 least enriched proteins in the interactome of (PR)_50_, compared with that of (YR)_50_ and the human proteome. (J) Amino acid occurrences in the 100 most enriched and 100 least enriched proteins in the interactome of (PR)_50_, compared with that of (YR)_50_, normalized to the human proteome. (K) Quantitative PCR of ribosomal RNA of HeLa cells treated with (PR)_20_ for the indicated periods. Expression levels were normalized to *ACTB*. N = 3. The error bar indicates the standard deviation. ∗∗: p < 0.01.

Differences in the mechanism of toxicity between poly(PR) and poly(GR) are expected to derive from their qualitatively and quantitatively different interactomes, which depend on the subcellular localization and valence of interaction. To investigate the manner of their protein-protein interactions, we performed interactome analysis using the TurboID-based proximity labeling method.^58^ TurboID fused to R_50_, (GR)_50_, (G_2_R_1_)_50_, (GR)_50_ mutant, or (PR)_50_, was expressed in HeLa cells. After induction of biotinylation, biotinylated proteins in the HeLa cells were visualized using AlexaFluor488-conjugated streptavidin (Figure 6A). Signals indicated that biotinylation of these proteins in close proximity was achieved. We also confirmed the biotinylation of the proteins by SDS-PAGE, followed by detection with horseradish peroxidase (HRP)-labeled streptavidin (Figure 6B). We purified the biotinylated proteins with streptavidin beads and analyzed them by quantitative liquid chromatography/mass spectrometry (LC/MS).^59^ We found that the interactome of (GR)_50_ was quite similar to that of R_50_, but different from that of (PR)_50_ (Figures 6C–6E). Importantly, the (PR)_50_ interactome was qualitatively similar to that of (G_2_R_1_)_50_ and (GR)_50_ mutants, but quantitatively different from them, indicating that the increased binding valence of proline determined the characteristics of the (PR)_50_ interactome (Figures 6D–6E, S9A, and S9B). We hypothesized that the strong inhibition of protein translation by (PR)_50_ might derive from the increased valence of protein-protein interactions. To test this hypothesis, we again performed proximity labeling analysis of (PR)_50_ and (YR)_50_, which localizes in the nucleolus in the same manner as (PR)_50_, but which exerts a less inhibitory effect on protein translation (Figure 4B). We found that both TurboID-(PR)_50_ and TurboID-(YR)_50_ exclusively biotinylated proteins in the nucleolus (Figures 6A and 6F), but that the signal intensities of many biotinylated nucleolar proteins were higher in the interactome of (PR)_50_ than in that of (YR)_50_ (Figure 6G).

In our previous study, we demonstrated that alternate insertions of proline into consecutive arginines confer a propensity for binding to acidic proteins. Therefore, we compared the isoelectric points (pI) of the 100 most and the 100 least enriched nucleolar proteins. The most enriched proteins tend to have slightly lower pIs when compared with the least enriched proteomes (Figure 6H). When we sought the longest acidic stretch in the proteins, we found that the 100 most enriched proteins contain significantly longer acidic stretches than the least enriched proteins (p = 0.00029) (Figure 6I). Amino acid occurrence in those proteins shows that acidic residues, but not aromatic residues were overrepresented in the poly(PR) interactome (Figure 6J). These results suggest that alternate insertions of proline have a more substantial impact on the interaction with acidic stretches than alternate insertions of tyrosine. STRING analysis of proteins with signal intensities that more than doubled in the (PR)_50_ interactome revealed that many of these proteins are involved in ribosomal RNA (rRNA) processing [GO: 0006364, False discovery rate (FDR) = 5.56×e−76] (Figures S9C and S10). We measured rRNA expression levels by quantitative PCR and confirmed that (PR)_20_ peptide treatment inhibited rRNA processing with increased pre-ribosomal RNA (Figure 6K). This suggests that the strong inhibitory effect of poly(PR) on protein translation was due in part to disturbance of rRNA processing.

## Discussion

Poly(PR) and poly(GR), produced from mutant *C9ORF72*, are toxic *in vitro* and *in vivo* and are thought to contribute to pathogenesis of ALS.^60^ However, it is unclear what accounts for differences in their localization and toxicity mechanisms. In this study, we found that poly(GR) binds strongly to a small number of molecules, whereas poly(PR) binds weakly to a large number of molecules. We expect that the rigidity that is a unique feature of proline, rather than the size of the side chain, contributes to achieving highly multivalent interactions because even though tyrosine has a larger side chain than proline, poly(YR) interacted with proteins in a less-multivalent manner (Figures 4D and 6G). Furthermore, proline efficiently and adequately separates the charges of arginines, which promotes the transition from the cytosol to the nucleolus. In contrast, glycine is flexible, and poly(GR) behaves like polyR and localizes to the cytoplasm. These biochemical features produce differences in localization and toxicity between poly(PR) and poly(GR).

Glycine is unique in structure. It has only a hydrogen atom as its side chain; thus, it is conformationally very flexible, allowing the main chain to adopt many conformations. Therefore, poly(GR) can easily achieve a good alignment with consecutive acidic sequences, as does polyR,^46^ and can form strong intermolecular interactions at a low molar ratio. Proline is also a unique amino acid in which the side chain is connected to the main chain twice, forming a five-membered, nitrogen-containing pyrrolidine ring. Because of this nature, proline cannot occupy many of the main chain conformations that all other amino acids can adopt; thus, proline often forms tight turns where the peptide chain must change its direction. The high steric hindrance and rigidity of proline strongly interfere with the movement of the main chain. Therefore, poly(PR) is less likely to align well with consecutive acidic groups and binds only partially, allowing it to bind weakly to a large number of molecules. Furthermore, poly(PR) prefers more linear structures because of the rigidity of proline (Figure 3A), which increases the probability of encountering a partner molecule to which it binds. Periodic insertions of proline into poly(GR) may interfere with strong interactions with acidic molecules, resulting in nucleolar localization. We speculate that the rigidity of proline rather than steric hindrance by the side chain contributes to multivalent interactions by insertions of proline, because (PR)_12_ binds to more molecules than other (XR)_12_ peptides that have side chains larger than that of proline (Figure 4D). This finding was further substantiated by performing proximity labeling-based interactome analysis of (PR)_50_ and bulky (YR)_50_ (Figure 6G). Amino acid occurrence in the interactome revealed that poly(PR) interacts with nucleolar proteins via electrostatic interactions, rather than cation-pi interaction, because acidic amino acids, but not aromatic amino acids were overrepresented in the interactome (Figure 6J).

MLOs are formed by LLPS of RNA and ribonucleoprotein, and arginine-rich peptides have a positive charge that facilitates their interaction with RNA and their incorporation into the MLO.^32^ Localization of proteins to the nucleolus also requires that basic amino acids form clusters locally.^39^ However, as we have shown, contiguous arginine sequences of a certain length (N ≥ 30) are trapped in the cytoplasm and do not migrate to the MLO, including the nucleolus. The molecular mechanism accounting for why R_50_ failed to localize to the nucleolus, remaining in the cytoplasm is still unclear, but we hypothesize that the extremely consecutive R has high binding energy and therefore binds tightly to cytoplasmic molecules just after being translated by the ribosome. Therefore, it cannot be transported into the nucleus. This hypothesis is also supported by the fact that the degree of macromolecular crowding around R_50_ was high (Figure S4E). When we separated the arginine charges with alternate insertions of amino acids, we observed that most of the GFP-(XR)_50_ migrated from the cytoplasm to the nucleolus. Since only poly(PR) acquired highly multivalent binding and no enhancement of multivalent binding was observed for poly(YR) or poly(QR) (Figure 4D), appropriate segregation of arginine charges, but not high multivalency in binding is required for localization to the nucleolus. In addition, there is an appropriate spacer/sticker ratio for localization to the nucleolus, and the ratio depends on how efficiently the inserting amino acid can separate the charges of arginines. For example, for proline, which strongly restricts the freedom of arginine, (PR)_50_ variants with a P:R ratio = 1:1 or even 1:2 localize to the nucleolus, but when the ratio of P:R = 2:1, the cluster of basic amino acids necessary for nucleolar incorporation is not formed, and affinity for the nucleolus is not formed, allowing it to freely diffuse in the cytosol. This mechanism is different from that of (P_1_R_3_)_16_, which is modestly trapped in the cytoplasm because of insufficient charge separation (Figures S7A and S7B). By contrast, single glycine insertion is not sufficient to separate the arginine charges and (GR)_50_ localizes to the cytoplasm like R_50_, whereas (G_2_R_1_)_50_, which is able to separate the charges sufficiently, localizes to the nucleolus. Proximity-dependent labeling also revealed that the degree of de-clustering of arginine charges determines the interactome of poly(XR) and differences in toxicity.

A lysine-to-arginine substitution in human RPL29 revealed that arginine has a more prominent impact on nucleolar cohesion. The comparison of polyK and polyR with different lengths indicated that arginines that are consecutive enough have a stronger propensity for static localization in the cytosol. These results suggest that although arginine and lysine are similar amino acids, they have fundamental differences. Arginine has intense binding capability because of the three-planer guanidinium ion, which enables simultaneous formation of cation-pi, pi-pi, and cation-anion contacts,^29^ whereas poly(PK) forms less viscous droplets than poly(PR) *in vitro.*^15^

Although inhibition of protein translation has been studied as an important aspect of R-DPR-mediated toxicity, the detailed molecular mechanism remains to be revealed. It reportedly binds directly to ribosomes and impairs their function,^61^ disrupting eIF1a-dependent pathways^54^ and affecting transcription.^34^ Because both poly(GR) and poly(PR) inhibit protein translation, translation inhibition could be a common feature of R-DPR-mediated toxicity. However, the degree of inhibition varied widely even among (PR)_50_, (MR)_50_, (QR)_50_, (TR)_50_, (YR)_50_, and (AR)_50_, which localize exclusively in the nucleolus. Comparison of the interactomes of (PR)_50_ and (YR)_50_ by proximity labeling revealed that (PR)_50_ binds to a group of molecules involved in rRNA processing in the nucleolus, and this may partially explain the strong inhibitory effect of poly(PR). However, cytosolic poly(GR) may inhibit protein translation via a different mechanism, and further studies are needed.

In conclusion, we revealed molecular mechanisms determining the subcellular localization and different toxicities of poly(PR) and poly(GR). This finding will contribute to development of specific inhibitors for R-DPR-mediated toxicity. Furthermore, the clarified roles of spacer amino acids will accelerate research in phase-separation and MLO-associated proteins.

### Limitations of the study

There are several limitations to this study. The first is that relatively short DPRs were used. That these may differ from biochemical features of naturally occurring DPRs with hundreds to thousands of repeats cannot be ruled out. Although even less than 50 repeats have been reported to contribute to the development of ALS with DPR pathology,^62^^,^^63^^,^^64^ the HREs longer than 200 are considered as very likely pathogenic.^41^ We confirmed that the localization of (PR)_200_ and (GR)_200_, which can be translated from 200 repeats is the same as that of 50 repeats. As previously reported, localization of R-DPRs with more than 1000 repeats is consistent with that of DPRs with 50 repeats that we examined here.^25^ The longer the repeat length, the greater the electrostatic force, so there is still a possibility that even poly(PR) will begin to localize to the cytoplasm above a certain threshold. Although our results clearly show that poly(PR) and poly(GR) localize differently up to 200 repeats, the subcellular localization of R-DPRs with several thousand repeats, as seen in ALS patients, requires further investigation. Another limitation of the study is that we obtained the data from immortalized cell lines.

## STAR★Methods

### Key resources table

**Table:** 

REAGENT or RESOURCE	SOURCE	IDENTIFIER
**Antibodies**		

B23/NPM1 antibody	Proteintech	10361-1-AP
CoraLite555-cconjugated B23/NPM1 monoclonal antibody	Proteintech	CL555-60096; RRID:AB_2919656
NONO antibody	Proteintech	11058-1-AP; RRID:AB_2152167
IPO7 Polyclonal antibody	Proteintech	28289-1-AP; RRID:AB_2881106
RPL5 antibody	Proteintech	29092-1-AP; RRID:AB_2881240
Anti-Puromycin Antibody, clone 12D10	Millipore	MABE343; RRID:AB_2566826
Anti-mouse IgG, HRP-linked Antibody	Cell Signaling Technology	Cat# 7076; RRID:AB_330924
Anti-rabbit IgG, HRP-linked Antibody	Cell Signaling Technology	Cat# 7074; RRID:AB_2099233
Goat anti-mouse IgG (H + L) antibody, Alexa Fluor 594	Molecular Probes	Cat# A-11020, RRID:AB_141974
Anti EIF3A	Proteintech	26178-1-AP; RRID:AB_2880413
Anti NAP1L4	Proteintech	16018-1-AP; RRID:AB_2150711
Anti EFI3M	Proteintech	11423-1-AP; RRID:AB_2246386
Anti-GFP pAb	MBL	Cat# 598; RRID:AB_591819
LMNB1 antibody	Proteintech	12987-1-AP; RRID:AB_2136290
Anti G3BP1	Proteintech	13057-2-AP; RRID:AB_2232034
Anti SRP54	Proteintech	11729-1-AP; RRID:AB_2194724
Anti CKAP4	Proteintech	16686-1-AP; RRID:AB_2276275
Anti DDX21	Proteintech	10528-1-AP; RRID:AB_2092705
Anti NOP2	Proteintech	10448-1-AP; RRID:AB_2282772
Anti NCL	Cell Signaling Technology	#14574
Anti-HA antibody beads	Wako	010-23083
Anti-alpha-tubulin mAb [10G10]	Wako	017-25031

**Chemicals, peptides, and recombinant proteins**		

Peptides, see Table S1		
Leptomycin B	Santa Cruz biotechnolgy	SC358688
Biotin	Tokyo Chemical Industry	B0463
Streptavidin-HRP	Proteintech	SA00001-0
Streptavidin, Alexa Fluor 488 conjugated	ThermoFisher	S11223
cOmplete protease inhibitor cocktail	RPCHE	11697498001
Poly(A) Polyadenylic acid	ROCHE	10108626001

**Critical commercial assays**		

Lipofectamine 2000 transfection reagent	Life Technologies	11668019
PEI MAX transfection grade linear Polyethyleneimine hydrochloride	Polsciences, Inc.	24765-1, CAS# 49553-93-7
Subcellular Protein Fractionation Kit for Cultured Cells	ThermoFisher	78840
Dynabeads MyOne Streptavidin C1	ThemoFisher	65001
4-20% miniprotean TGX precast gel	Bio-rad	#4561096
Laemmli sample buffer	Bio-rad	#1610737
ECL Select Western Blotting Detection Reagent	GE Healthcare	RPN2235

**Deposited data**		

Mendeley Data	https://doi.org/10.17632/6jk2sjp53j.1	https://data.mendeley.com/drafts/6jk2sjp53j

**Experimental models: Cell lines**		

HeLa cells	RIKEN BRC	RCB3680
HEK293T cells	Clontech	632180
NSC34 cells	From Dr. Neil Cashman (UBC, Canada)	Cat#N/A; RRID: CVCL_D356;

**Oligonucleotides**		

TAMRA-rA15	This study	N/A
Human Actin B forward (CATGTACGTTGCTATCCAGGC)	This study	N/A
Human Actin B reverse (CTCCTTAATGTCACGCACGAT)	This study	N/A
Huan pre-ribosomal RNA forward (GAACGGTGGTGTGTCGTTC)	This study	N/A
Huan pre-ribosomal RNA reverse (GCGTCTCGTCTCGTCTCACT)	This study	N/A
Human 28S rRNA forward (AGAGGTAAACGGGTGGGGTC)	This study	N/A
Human 28S rRNA reverse (GGGGTCGGGAGGAACGG)	This study	N/A
Human 18S rRNA forward (GATGGTAGTCGCCGTGCC)	This study	N/A
Human 18S rRNA reverse (GCCTGCTGCCTTCCTTGG)	This study	N/A

**Recombinant DNA**		

See Table S2		

**Software and algorithms**		

NoD	Scott et al.^40^	http://www.compbio.dundee.ac.uk/www-nod/index.jsp
FIJI	NIH	https://fiji.sc/
EzColocalization	Stauffer et al.^65^	http://sites.ImageJ.net/EzColocalization/plugins/
SPSS ver.28	IBM	https://www.ibm.com/analytics/spss-statistics-software
ANACONDA	Anaconda.Inc	https://docs.anaconda.com
Seaborn 0.11.2	Waskom^66^	https://seaborn.pydata.org
GROMACS	Kumari et al.^49^	https://gromacs.bioexcel.eu
AVOGADRO	Hanwell et al.^67^	https://avogadro.cc
ZEN2.3	Zeiss, Göttingen, Germany	https://www.zeiss.com/microscopy/int/products/microscope-software/zen.html

**Others**		

Amicon 10 kDa spin column	Merck	800010
4-well chamber slide glass	Matsunami	SCS-N04
4-well chamber cover glass	Matsunami	SCC-004
Immobilon-P	Millipore	IPVH00010

### Resource availability

#### Lead contact

Further information and requests for resources and reagents should be directed to the lead contact, Kohsuke Kanekura (kanekura@tokyo-med.ac.jp).

#### Materials availability

Further information and requests for resources and reagents will be fulfilled by the lead contact author.

### Experimental model and subject details

#### Cell line

HeLa cells were obtained from RIKEN BRC. HEK293 cells were purchased from Clontech. NSC34 cells were provided by Dr. Neil Cashman.^68^ These cells were maintained at 37°C with 5% CO_2_ in a humidified incubator. Both cell lines were cultured in Dulbecco’s Modified Eagle Medium (DMEM, Gibco) supplemented with 10% fetal bovine serum (FBS) and penicillin/streptomycin (100 units/mL) (Sigma, P4333). These cell lines were periodically confirmed to be free of mycoplasma.

### Method details

#### Gene synthesis and expression

cDNA constructs coding repetitive amino acid sequences were designed not to repeat the same codons so as to avoid unexpected RAN translation. (PR)_50_, (P_4_R_4_)_12_, (P_8_R_8_)_6_, (P_16_R_16_)_3_, (GR)_50_, (G_16_R_16_)_3_, and (GR)_50_ mutant sequences were synthesized by GeneArt Gene Synthesis (Thermo Fisher Scientific) as BglII-EcoRI fragments in pMA vector (Thermo Fisher Scientific). cDNAs were then subcloned into pEGFP-C1 vectors at BglII-EcoRI sites. Other synthetic DPR constructs were chemically synthesized and subcloned into pcDNA3.1-N-EGFP at BamHI-EcoRI sites by Genscript. TurboID cDNA was also synthesized by Genscript. Transfection was performed with Lipofectamine 2000 (Thermo Fisher Scientific) unless otherwise mentioned. Immunoblot analyses confirmed expressions of all the (XR)_50_ constructs (Figure S11).

#### Immunoprecipitation and immunoblot analyses

All HA-tagged (XR)_12_ peptides were chemically synthesized by Genscript with purity >85%, and trifluoroacetic acid was substituted with acetic acid. For immunoprecipitation, 1 nmol of HA peptide or each HA-tagged (XR)_12_ peptide was mixed with lysates of HEK293 cells (200 μg/sample). IP was performed with IP buffer [150 mM NaCl, 20 mM HEPES (pH7.4), 1 mM EDTA, 0.5% Triton X-100 and cOmplete protease inhibitor cocktail (Roche)] and 10 μL of anti-HA antibody beads (Wako). After rotation for 4 h at 4°C, beads were centrifuged and washed 4x with IP buffer, followed by boiling with 2× Laemmli sample buffer (Bio-rad) at 95 °C for 5 min. Extracted proteins were subjected to SDS-PAGE using 4–20% precast gels (Bio-Rad) and transferred to Immobilon membranes (Millipore) using a semi-dry blotting system. To block non-specific signals, membranes were incubated with BlockingOne reagent (Nacalai) for 1 h at room temperature. Then, membranes were probed with the following antibodies: B23/NPM1 antibody (Proteintech, 10361-1-AP), NONO antibody (Proteintech, 11058-1-AP), IPO7 antibody (Proteintech, 28289-1-AP), RPL5 antibody (Proteintech, 29092-1-AP), EIF3A antibody (Proteintech, 26178-1-AP), NAP1L4 antibody (Proteintech, 16018-1-AP), EIF3M antibody (Proteintech, 11423-1-AP) (all antibodies were diluted at 1:1000 and probed overnight at 4°C). Signals were visualized using HRP-conjugated anti-rabbit secondary antibody (Cell Signaling Technology, #7074, 1:5000) and ECL Select Western Blotting Detection Reagent (GE Healthcare). Signals were detected using a ChemiDoc Touch imaging system (Bio-rad). Uncropped images of immunoblot analyses were shown in Figure S12.

#### Fractionation and immunoblot analyses

Fractionation was performed using Subcellular Protein Fractionation Kit for Cultured Cells (Thermo Fisher Scientific), following the manufacturer’s instructions. HEK293 cells were plated on a 10 cm dish the day before transfection. Cells were transfected with poly(XR) constructs using Lipofectamine 2000 (Thermo Fisher Scientific). Twenty-four h after transfection, cells were trypsinized and centrifuged at 500 ×g for 5 min and they were resuspended with ice-cold PBS. Cell pellets were transferred to 1.5-mL microcentrifuge tubes and centrifuged at 500 ×g for 5 min and supernatants were aspirated. Ice-cold CEB buffer containing protease inhibitors was added to the pellets on ice. After 10 min incubation with gentle mixing on ice, cells were centrifuged at 500 ×g for 5 min, supernatant fractions (cytosol) were collected. The remaining pellets containing nuclei were lysed in sample buffer. The cytosolic fraction and nuclear fractions were subjected to SDS-PAGE followed by immunoblot analyses. Membranes were probed with the following antibodies: GFP antibody (MBL, #598, 1:3000), LMNB1 antibody (Proteintech, 12987-1-AP, 1:3000), alpha-tubulin antibody (Wako, 017-25031, 1:3000). The signals were visualized by HRP-conjugated anti-rabbit secondary antibody (Cell Signaling Technology, #7074, 1:5000) or HRP-conjugated anti-mouse secondary antibody (Cell Signaling Technology, #7076, 1:5000).

#### LLPS and fluorescence recovery after photo bleaching (FRAP) analysis

For observation of LLPS of R_12_, (PR)_12_ or (GR)_12_ and poly-rA, each peptide (final concentration 100 μM) and poly-rA (final concentration 0.5 mg/mL) containing 100 nM TAMRA-rA_15_ (Fasmac) were mixed. Droplets were plated on chambered cover glasses and covered with Silicon oil AP100 (Sigma). Droplets were observed using an LSM-710 confocal microscope (Carl Zeiss). FRAP analyses were also performed with an LSM-710 confocal microscope using a lens of N.A. = 1.2. For FRAP analysis, HeLa cells were seeded on 4-well chambered cover glasses (Matsunami) at 3×10^4^ cells/500 μL/well the day before transfection. Twenty-four h after transfection, FRAP analyses were performed with an LSM-710 confocal microscope using the same settings. Data were analyzed with ZEN software (Carl Zeiss).

#### Critical salt concentration

To determine the critical salt concentration, 50 μM of (PR)_20_, (GR)_20_ or (GR)_20_ mutant peptides were mixed with 50 μM of KAPB2-H8 peptide in a phase-separation buffer (10 mM HEPES, pH 7.4) containing different concentrations of NaCl from 0 to 400 mM. Turbidity was measured by absorbance at 600 nm using NanoDrop One (Thermo Fisher Scientific).

#### Phase diagram of R-DPRs with different ratios of KAPB2-H8

To evaluate the multivalency of interactions, each R-DPR at the indicated concentration was mixed with different concentrations of KAPB2-H8 peptide in phase separation buffer [10 mM HEPES (pH7.4) and 100 mM NaCl]. Turbidity was measured with absorbance at 600 nm using NanoDrop One (Thermo Fisher Scientific).

#### Evaluation of macromolecular crowding in LLPS droplets using a CRONOS sensor

A Förster-resonance energy transfer (FRET)-based fluorescent biosensor CRONOS (crowding sensor with mNeonGreen and mScarlet-I) was used to evaluate macromolecular crowding in LLPS droplets consisting of R-DPRs and KAPB2-H8.^47^ To obtain recombinant CRONOS-KAPB2-H8, *E. coli* BL21 strain (New England Biolabs) was transformed with pET28a-CRONOS-KAPB2-H8 and cultured overnight on an LB agar plate containing kanamycin. A single colony was picked and cultured in 2× YT growth medium at 37 °C until the OD_600_ reached 1.0. Expression of CRONOS-KAPB2-H8 was induced by adding 1 mM isopropyl β-D-thiogalactopyranoside (IPTG), and cells were cultured overnight at 25 °C. The *E. coli* expressing recombinant CRONOS-KAPB2-H8 was pelleted by centrifugation at 4000 ×g for 10 min and sonicated in lysis buffer [50 mM Tris-HCl (pH 7.4), 500 mM NaCl, 1% Triton X-100, cOmplete protease inhibitor cocktail (Roche), 0.1 mg/mL lysozyme (Wako chemical)]. Recombinant CRONOS-KAPB2-H8 was purified with a His60 Ni Gravity column purification Kit (Clontech) following the manufacturer’s protocol. After elution with 500 mM imidazole, recombinant CRONOS-KAPB2-H8 was dialyzed overnight with PBS using Slide-a-Lyzer dialysis cassette (Thermo Fisher Scientific). The concentration of purified CRONOS-KAPB2-H8 was measured using NanoDrop One (Thermo Fisher Scientific), and then the protein was frozen at −80 °C until use.

To measure macromolecular crowding in LLPS droplets, (GR)_20_, (GR)_20_ mutant or (PR)_20_ peptide (final 50 μM) was mixed with KAPB2-H8 peptide (final 100 μM) in phase separation buffer [10 mM HEPES (pH 7.4) and 100 mM NaCl] containing 1 μM CRONOS-KAPB2-H8. FRET ratios in droplets were determined using an FLUOVIEW FV-10i confocal microscope (Olympus). Images were analyzed with ImageJ software.

#### Immunofluorescence and confocal imaging

HeLa cells were seeded on 4-well chambered slide glasses (Matsunami) at 5×10^4^ cells/500 μL/well the day before transfection. Twenty-four h after transfection, cells were fixed with 4% formalin-PBS for 15 min at room temperature for imaging analysis. For identification of the nucleolus, endogenous NPM1 was visualized by CoraLite555-conjugated anti-NPM1 antibody (1:2500 dilution) (Proteintech). For visualization of the nuclear speckles, SRSF1-mCherry was coexpressed. For evaluation of nucleocytoplasmic export, transfected cells were incubated with 100 nM leptomycin B for 2 h before fixation. For the SUnSET assay, transfected cells were pulsed in the presence of 2 μM puromycin for 1 h before fixation. Fixed cells were washed with PBS and permeabilized with 0.5% Triton X-100 containing IP buffer for 5 min. After blocking with 50% diluted BlockingOne (Nacalai) for 1 h at room temperature, cells were incubated with anti-puromycin antibody (Millipore, MABE343, 1:500) diluted in IP buffer for 1 h at room temperature. Then, cells were washed 4x with IP buffer, followed by incubation with AlexaFluor 488-conjugated anti-mouse IgG (Molecular Probes, A-21202, 1:500). After 4 washes with IP buffer, slides were mounted with ProLong Gold Antifade Mountant with DAPI (Thermo Fisher Scientific). Confocal imaging was performed with an FLUOVIEW FV-10i confocal microscope (Olympus). Images were analyzed using ImageJ software.

#### PCC analysis

PCC analysis was performed using EzColocalization, an open-source plugin for ImageJ, to evaluate colocalization in confocal imaging.^65^ The data obtained from PCC analysis were imported into Spyder (Python v3.8) in Anaconda and plotted using Seaborn.^66^

#### Proximity labeling and quantitative liquid chromatography/mass spectrometry

TurboID-based proximity labeling was performed as previously reported.^30^^,^^58^ Briefly, HeLa cells were plated on a 15-cm dish the day before transfection. Cells were transfected with TurboID-R_50_, TurboID-(GR)_50_, TurboID-(G_2_R_1_)_50_, TurboID-(GR)_50_ mutant, TurboID-(PR)_50_ or TurboID-(YR)_50_ using PEI MAX reagent (Polysciences). Twenty-four h after transfection, cells were reacted with 50 μM biotin for 2 h. Cells were washed 4× with ice-cold PBS, followed by lysis using RIPA buffer containing cOmplete protease inhibitor cocktail (Roche). Excess biotin was removed by 3× ultrafiltration using an Amicon Ultra filter (cutoff 10K). To purify biotinylated proteins, processed lysates were incubated with Dynabeads MyOne streptavidin beads (Thermo Fisher Scientific) in PBS for 2 h at 4°C. Beads were sequentially washed with RIPA buffer, 1 M KCl, 0.1 M Na_2_CO_3_, 2 M urea in 10 mM Tris-HCl (pH 7.5), and RIPA buffer. We used three biological replicates per construct. Samples were subjected to quantitative liquid chromatography/mass spectrometry.^59^ Briefly, biotinylated proteins on the beads were reduced with 10 mM TCEP at 100°C for 10 min, alkylated with 50 mM iodoacetamide at room temperature for 45 min, and then digested on-beads with Trypsin/Lys-C Mix (Promega) at 37°C for 12 h. The resulting peptides were analyzed with an Orbitrap Fusion Lumos mass spectrometer (Thermo Fisher Scientific) combined with an UltiMate 3000 RSLC nano-flow HPLC (Thermo Fisher Scientific). Peptides were enriched with a μ-Precolumn (0.3 mm i.d. × 5 mm, 5 μm, Thermo Fisher Scientific) and separated on an AURORA column (0.075 mm i.d. × 250 mm, 1.6 μm, Ion Opticks Pty Ltd, Australia) using a two-step gradient, 2–40% acetonitrile for 110 min, followed by 40–95% acetonitrile for 5 min in 0.1% formic acid. Analytical parameters of the Orbitrap Fusion Lumos were set as follows: Resolution of full scans = 50,000, Scan range (*m*/*z*) = 350–1500, Maximum injection time of full scans = 50 msec, AGC target of full scans = 4 × 10^5^, Dynamic exclusion duration = 30 s, Cycle time of data dependent MS/MS acquisition = 2 s, Activation type = HCD, Detector of MS/MS = Ion trap, Maximum injection time of MS/MS = 35 msec, AGC target of MS/MS = 1 × 10^4^. MS/MS spectra were searched against the *Homo sapiens* protein sequence database in SwissProt using Proteome Discoverer 2.5 software (Thermo Fisher Scientific), in which peptide identification filters were set at a “false discovery rate <1%”. Label-free relative quantification analysis for proteins was performed with default parameters of Minora Feature Detector node, Feature Mapper node, and Precursor Ions Quantifier node in Proteome Discoverer 2.5 software.

#### Heatmap of interactome

MetaboAnalyst (ver. 5.0) [PMID: 34019663] was used to visualize a heatmap of proteins identified by proximity labeling. Signal intensities of each protein were normalized with the sum of total peptide score. Normalized abundance data were further processed. Features with any missing values were excluded, and quantile normalization for sample normalization and auto-scaling (*Z* score) for each feature was conducted. Averaged data for each group were clustered with Pearson correlation, and features showing high variances among groups within the top 25 were visualized. Colors red, blue, and white indicate higher, lower, and average.

#### Gene ontology analysis

STRING interaction network of nucleolar proteins with signal intensities that were at least doubled in the interactome of (PR)_50_ when compared with that of (YR)_50_ were analyzed with STRING (www.string-db.org)^69^ for gene ontology analysis.

#### Quantitative PCR of rRNA

HEK293 cells were cultured in the presence of 10 μM of (PR)_20_ peptide for 8 or 24 h. Total RNA was extracted with TRIzol reagent (Invitrogen) following the manufacturer’s protocol. cDNA was synthesized from 1 μg of total RNA using a Superscript III First-Strand Synthesis System (Thermo Fisher Scientific). Expression levels of pre-ribosomal RNA, 28S rRNA, 18S rRNA, and 5S rRNA were quantified with DyNAmo ColorFlash SYBR Green qPCR Kit (Thermo Fisher Scientific). Quantitative PCR was performed with QuantStudio Real-Time PCR system (Thermo Fisher Scientific). PCR was carried out with the following conditions: initial denaturation at 94 °C for 3 min, and 45 cycles of 94 °C for 15 s, 60 °C for 1 min. Relative expression levels were normalized against *ACTB*.

#### Computational identification of (X-R/K) repeats in natural proteins

In order to extract periodically positively charged motifs and subcellular localization signatures from the human proteome, we performed data mining of protein sequences obtained from the reviewed human proteome (UniProt accession AUP000005640). We analyzed protein sequences and subcellular localization data by importing human proteome data from UniProt into Spyder (Python v3.8) in Anaconda (https://docs.anaconda.com). First, we searched proteins harboring X-R/K repeats with repeat lengths 5 or longer. Out of 20,386 human proteins, we found 797 proteins containing X-R/K repeats with repeat lengths of at least 5. Next, we counted the number of each amino acid in the X-R/K repeats, and compared their frequencies with their general occurrences in the entire human proteome. Hypergeometric p values were calculated with the web server https://systems.crump.ucla.edu/hypergeometric/index.php. We also accessed the UniProt API (https://www.uniprot.org/help/programmatic_access) to obtain xml files of each protein with X-R/K repeats and collected subcellular localization information. Among repeat-containing proteins, 112 proteins with X-R/K repeats 5 or longer and 4 proteins containing 10 or longer repeats were excluded due to lack of xml or localization data. We also collected localization information for 16,810 proteins (no data: 3576 proteins) in the whole proteome.

#### Data mining

Data mining was performed to extract acidic stretches from the nucleolar proteome. We analyzed protein sequence data based on Anaconda by importing data from Uniprot into Spyder (Python 3.7.3) in Anaconda (https://docs.anaconda.com/anaconda/).

First, the UniProt application programming interface (API) was used to pick up proteins localized to the nucleolus from the (PR)_50_ and (YR)_50_ interactomes detected by LC/MS, respectively. Next, sequence information was extracted from Uniprot for the 100 most and 100 least enriched proteins in (PR)_50_ compared to (YR)_50_, and the length of the longest consecutive acidic amino acid (D/E) sequence was examined. Lengths of contiguous D/E sequences were similarly examined in the total human proteome (20,327 proteins). Frequencies of occurrence of each amino acid in these 200 nucleolar proteins were also examined and compared with frequencies in the entire human proteome (20,327 proteins).

#### MD simulation

(PR)_12_ and (GR)_12_ were modeled using AVOGADRO software and studied at pH 7, with both N-terminal proline/glycine and C-terminal arginine residues being protonated. MD simulation was performed using GROMACS 2022.1 and the OPLS-AA/L all-atom forcefield was used.^67^^,^^70^^,^^71^ A cubic box (20 × 20 × 20 Å^3^) with periodic boundary conditions applied in all directions was used for all simulations. The entire system was solvated with an explicit SPC (Single Point Charge) water model and neutralization was achieved by addition of chloride ions. First, we performed energy minimization with the steepest decent method to reach the maximum force <1,000 kJ/mol. We pre-equilibrated the systems for 100 ps at a constant temperature of 300 K using the V-rescale coupling method in a canonical (NVT) ensemble and 100 ps at a constant pressure of 1 bar using the Parrinello–Rahman coupling method^72^ in the isothermal-isobaric (NPT) ensemble. Finally, we carried out a 30-ns MD simulation for each peptide, and we used the data from 10 ns to 30 ns. Simulation trajectories were visualized with Grace (https://plasma-gate.weizmann.ac.il/Grace/). Structural deviations along the trajectories were evaluated using root-mean-square deviations (RMSD) of the peptide. The structural compactness was evaluated by radius of gyration of the peptide.

### Quantification and statistical analysis

Statistical analyses were performed by one-way ANOVA with the Dunnett’s test or Tukey’s test for comparing 3 or more groups and unpaired, two-tailed t tests to compare 2 groups using SPSS software ver. 28 (IBM). Unless otherwise mentioned, all experiments were performed in triplicate.

## Data Availability

Proteomic data, raw data of confocal images, and immunoblot images are publicly available via Mendeley data (https://doi.org/10.17632/6jk2sjp53j.1) as the date of publication. This paper does not report original code. Any additional information required to reanalyze the data reported in this paper is available from the lead contact upon request.

## Supplemental information

Document S1. Figures S1–S12 and Table S1

Table S2. List of recombinant DNA, related Figures 1–6
