# Supplementary material for: Differential toxicity and localization of arginine-rich C9ORF72 dipeptide repeat proteins depend on de-clustering of positive charges
Source: iScience. 2023 May 25;26(6):106957. doi: 10.1016/j.isci.2023.106957 (PMC10275993; doi:10.1016/j.isci.2023.106957)
Supplement: Document S1. Figures S1–S12 and Table S1 [file mmc1.pdf]

iScience, Volume *26*

Supplemental information

Differential toxicity and localization of arginine-rich *C9ORF72* dipeptide repeat proteins depend on de-clustering of positive charges

Tamami Miyagi, Koji Ueda, Masahiro Sugimoto, Takuya Yagi, Daisuke Ito, Rio Yamazaki, Satoshi Narumi, Yuhei Hayamizu, Hiroshi Uji-i, Masahiko Kuroda, and Kohsuke Kanekura

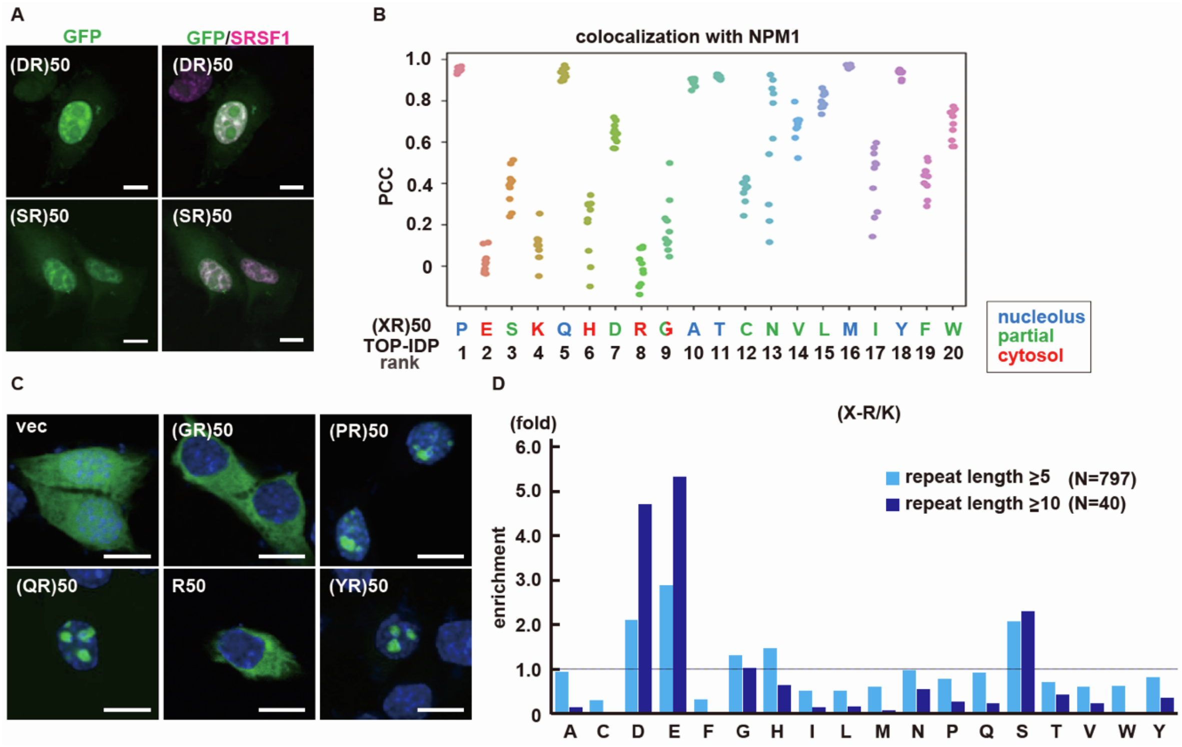

Figure S1. The (XR)$_{50}$ facilitates distribution to membrane-less organelles, related to Figure 1.

(A) Representative images of HeLa cells expressing GFP-(DR)$_{50}$ or GFP-(SR)$_{50}$. The nuclear speckles were identified by serine/arginine-rich splicing factor 1 (SRSF1)-mCherry. Scale bar: 10 μm.

(B) The Pearson correlation coefficient (PCC) of GFP-(XR)$_{50}$ and NPM1. Ten cells/each were evaluated using the ImageJ EzColocalization Plugin. TOP-IDP is used as the disorder propensity scale, and amino acids are arranged in order of TOP-IDP scale. Blue colored: exclusively nucleolar. Green colored: partially nucleolar. Red colored: exclusively cytosolic.

(C) Representative images of NSC34 cells expressing GFP vector, GFP-R$_{50}$, GFP-(PR)$_{50}$, GFP-(GR)$_{50}$, GFP-(QR)$_{50}$ or GFP-(YR)$_{50}$. Nuclei were visualized with DAPI. Scale bar: 20 μm.

(D) Amino acid occurrences in (X-R/K) repeats in the human proteome.

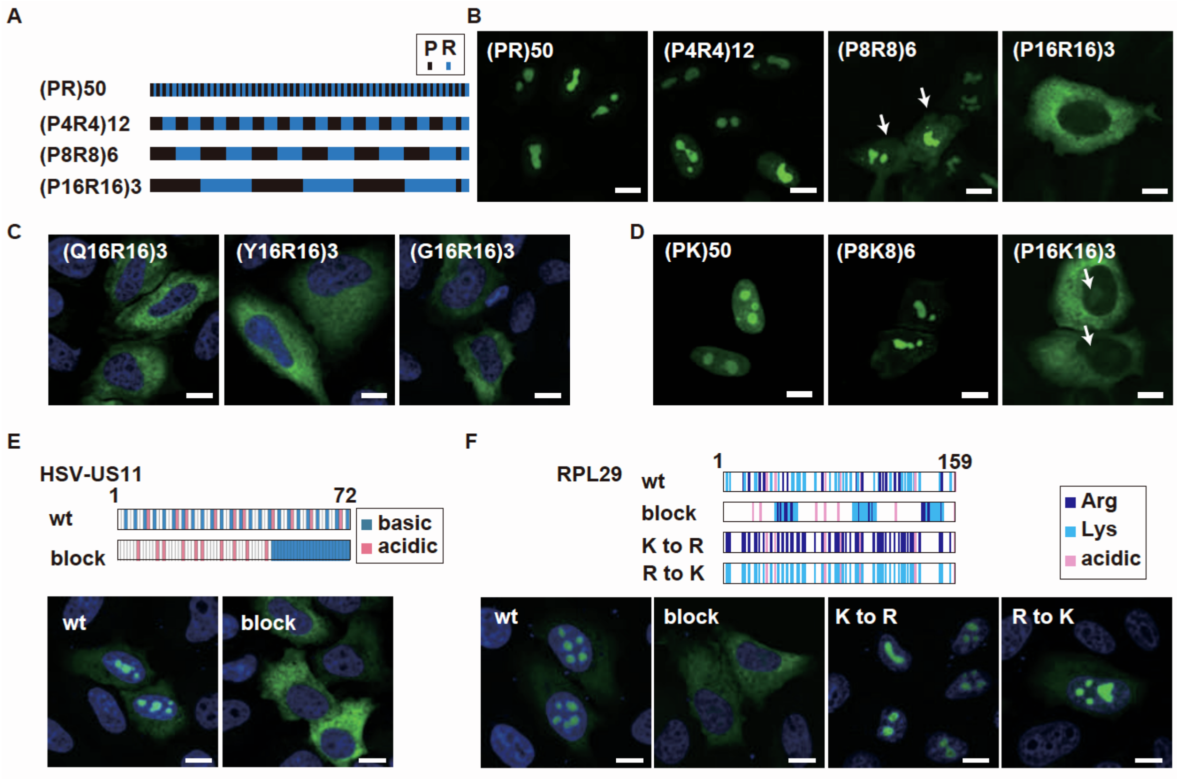

Figure S2. The alternate structure of R-DPR is important for nucleolar distribution, related to Figure 2.

(A) Diagram of ratio variants of $(PR)_{50}$ variants with different arginine distributions. P_2R_2 was fused to the C-terminus of $(P_4R_4)_{12}$, $(P_8R_8)_6$ and $(P_{16}R_{16})_3$ to equalize the number of arginines at 50.

(B) Representative images of HeLa cells expressing GFP-$(PR)_{50}$ variants. Scale bars: 10 μm. White arrows indicate the faint cytosolic signal.

(C) Representative images of HeLa cells expressing GFP-$(Q_{16}R_{16})_3$, GFP-$(Y_{16}R_{16})_3$, or GFP-$(G_{16}R_{16})_3$. Scale bar: 10 μm.

(D) Representative images of HeLa cells expressing GFP-$(PK)_{50}$ variants. Scale bar: 10 μm.

(E) Representative images of HeLa cells expressing GFP-US11-wt or GFP-US11-block. Scale bar: 10 μm.

(F) Representative images of HeLa cells expressing GFP-RPL29-wt, GFP-RPL29-block, GFP-RPL29-K to R mutant or GFP-RPL29-R to K mutant. Scale bar: 10 μm.

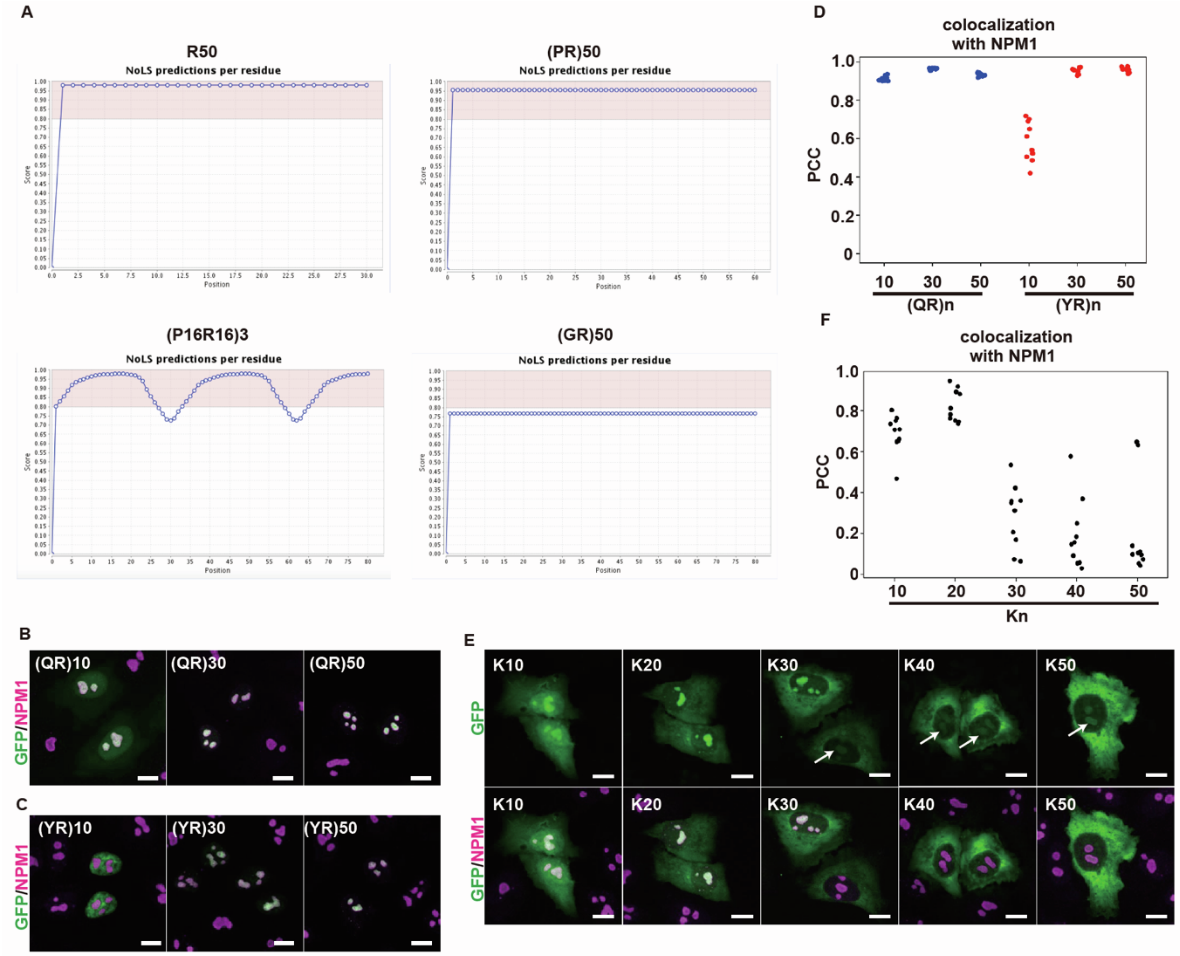

Figure S3. Repeat-length-dependent nucleolar localization of basic peptides, related to Figure 2.

(A) Evaluation of R_{50}, $(PR)_{50}$, $(P_{16}R_{16})_3$, and $(GR)_{50}$ as NoLS by the NoD webserver. Each amino acid sequence was analyzed by NoD webserver whether it was recognized as NoLS or not. The amino acid sequences in the pink area (>0.8) were recognized as NoLS.

(B) Representative images of HeLa cells expressing GFP-poly(QR) with different repeat lengths. The nucleolus was visualized by CoraLite555-conjugated anti-NPM1 antibody. Scale bars: 10 μm.

(C) Representative images of HeLa cells expressing GFP-poly(YR) with different repeat lengths. Scale bar: 10 μm.

(D) PCCs of each GFP-poly(QR) or poly(YR) and NPM1. Ten cells/each were evaluated.

(E) Representative images of HeLa cells expressing GFP-polyK with different lengths. Scale bar: 10 μm.

(F) PCCs of each GFP-polyK and NPM1. Ten cells/each were evaluated.

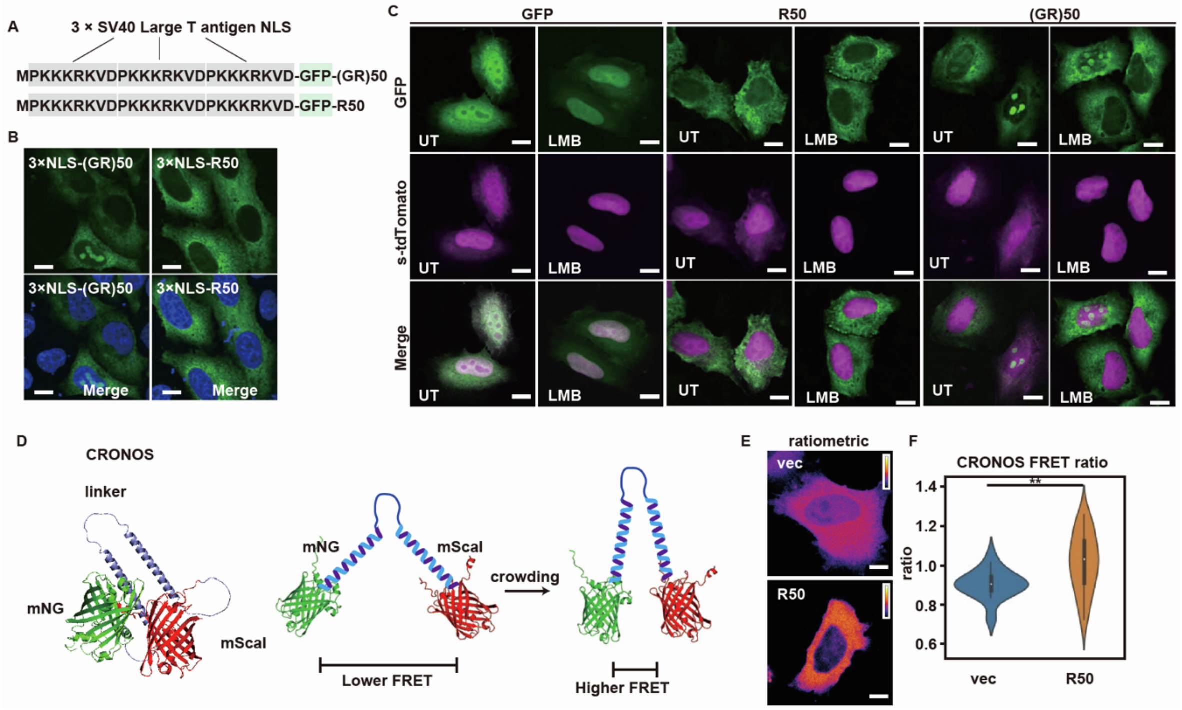

Figure S4. PolyR and poly(GR) statically localize to the cytosol, related to Figure 2.

(A) The sequence of 3×NLS of the SV40 large T antigen fused to the N-terminus of GFP-$(GR)_{50}$ or GFP-R_{50}.

(B) Representative images of HeLa cells expressing 3×NLS-GFP-$(GR)_{50}$ or 3×NLS-GFP-R_{50}. Nuclei were visualized with DAPI. Scale bars: 10 μm.

(C) Representative images of HeLa cells co-expressing GFP, GFP-R_{50} or GFP-$(GR)_{50}$ and s-tdTomato in the presence or absence of 100 nM leptomycin B (LMB). Scale bar: 10 μm.

(D) Structure of the CRONOS sensor. The structure was speculated with AlphaFold2 [1]. mNeonGreen is indicated in green and mScarlet-I is in red.

(E) Representative ratiometric images of HeLa cells expressing a macromolecular crowding sensor CRONOS vector or CRONOS-R_{50}. Scale bar: 10 μm.

(F) Violin plot indicating the FRET ratios of CRONOS and CRONOS-R_{50}. N=27 cells /each. **: p<0.01

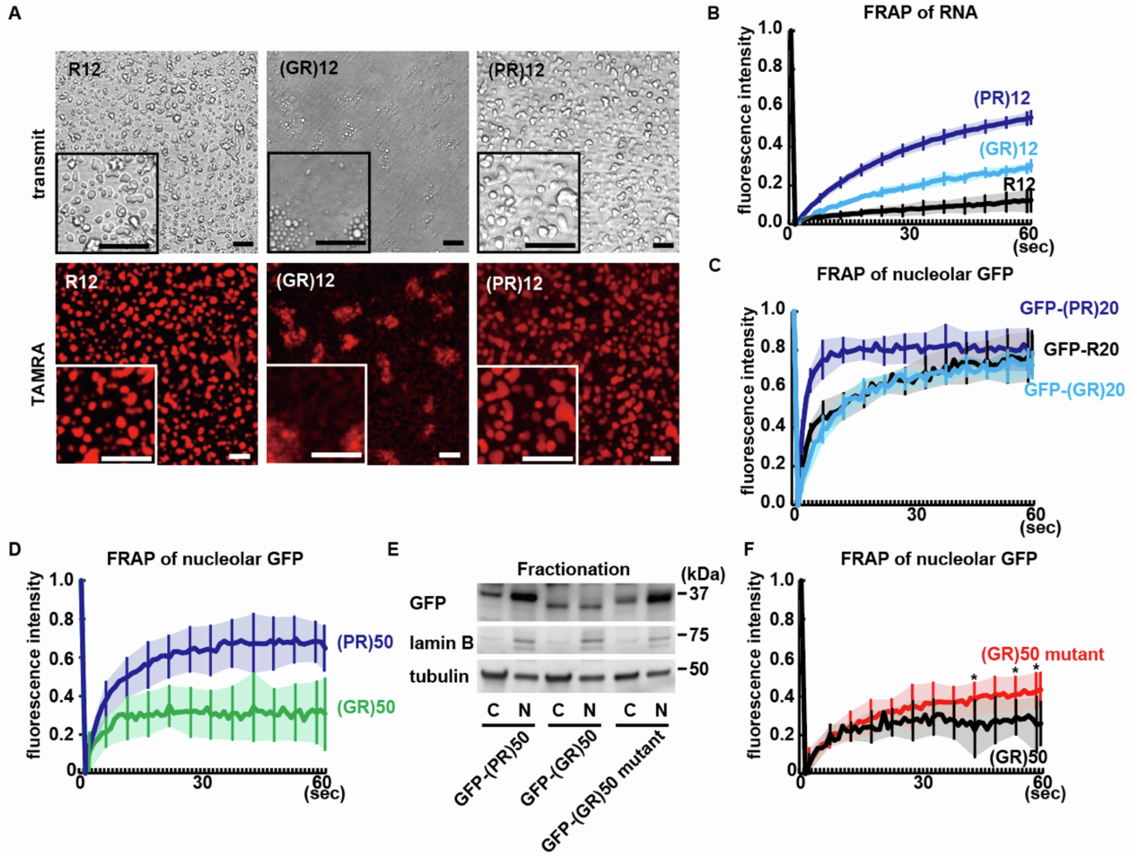

Figure S5. Insertion of proline loosens the interaction between arginine residues and acidic biomolecules, related to Figure 3.

(A) Representative images of LLPS droplets consisting of poly-rA and indicated peptides. Scale bar: 10 μm.

(B) Limited FRAP recovery of TAMRA-RNA phase-separated with R_{12} and $(GR)_{12}$, compared to that of $(PR)_{12}$. Mean values and standard deviations from 6 independent analyses are shown.

(C) Limited FRAP recovery of GFP-R_{20} and GFP-$(GR)_{20}$ in the nucleolus, compared to GFP-$(PR)_{20}$. Mean values and standard deviations from 10 cells are shown.

(D) Limited FRAP recovery of GFP-$(GR)_{50}$ in the nucleolus, compared to GFP-$(PR)_{50}$. Mean values and standard deviations from 10 cells are shown.

(E) Immunoblot of cytosolic fractions (C) and nuclear fractions (N) of HeLa cells expressing GFP-$(PR)_{50}$, GFP-$(GR)_{50}$ or GFP-$(GR)_{50}$ mutant. Lamin B is a nuclear marker and tubulin is a cytosolic marker, respectively.

(F) Partial rescue of FRAP recovery of GFP-$(GR)_{50}$ in the nucleolus by periodic insertions of proline. *: $p<0.05$.

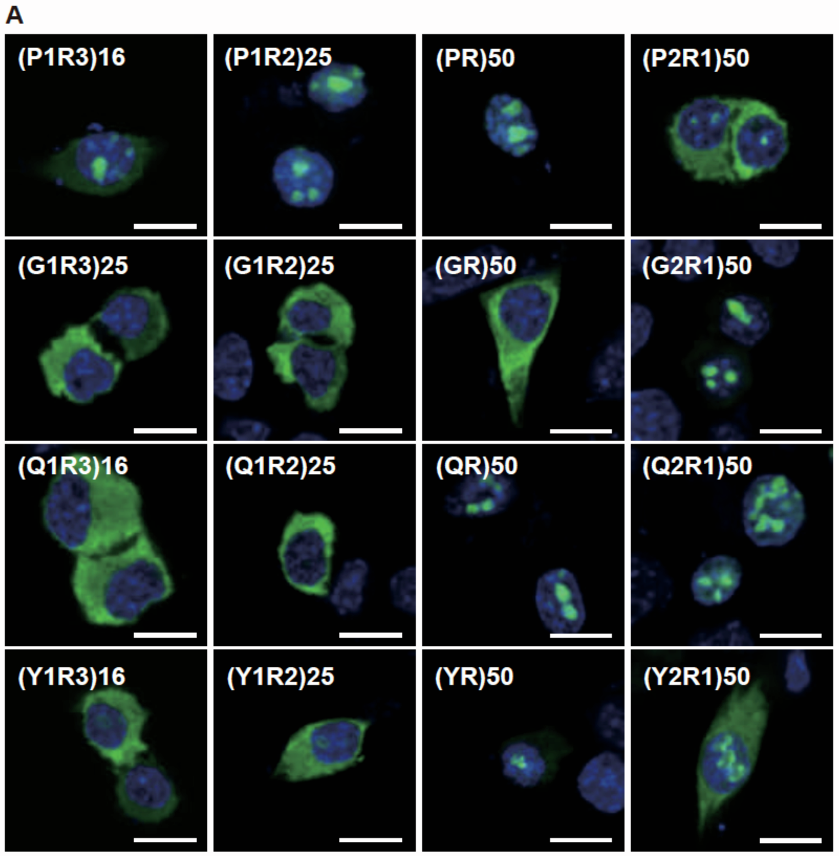

Figure S6. Appropriate segregation of arginine charges is essential for nucleolar incorporation in NSC34 cells, related to Figure 5.

(A) Representative images of NSC34 cells expressing GFP-$(XR)_{50}$ ratio variants. Nuclei were visualized with DAPI. Scale bar: 20 μm.

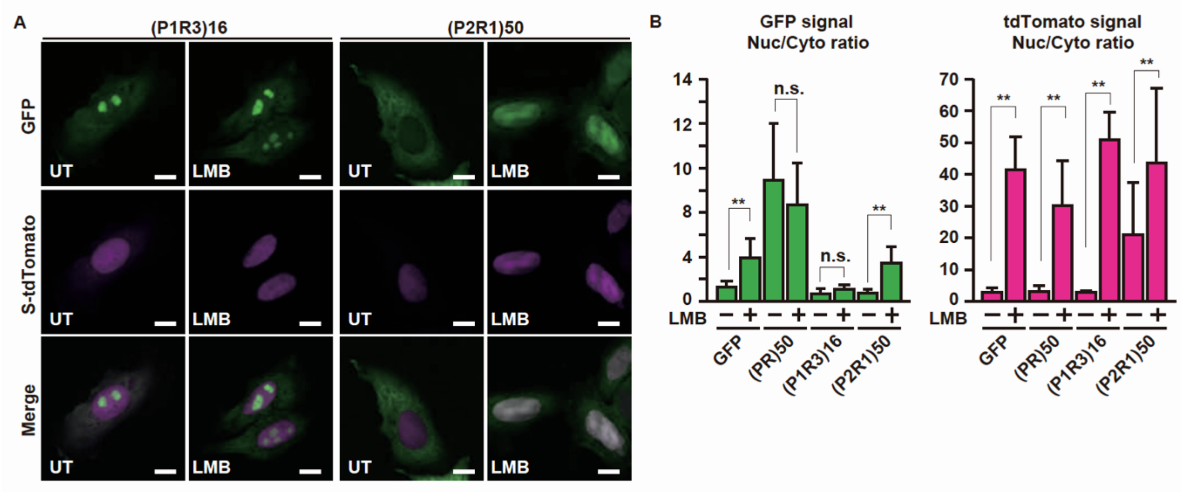

Figure S7. The ratio of proline and arginine determines the degree of nucleocytoplasmic shuttling of GFP-(PR)$_{50}$ variants, related to Figure 5.

(A) Representative images of HeLa cells co-expressing GFP-(P_1R_3)$_{16}$ or GFP-(P_2R_1)$_{50}$ and s-tdTomato in the presence or absence of 100 nM leptomycin B (LMB). Scale bars: 10 μm.

(B) The ratio of signal intensity of GFP in the nucleolus/cytosol (Nuc/Cyto). N=6~12 cells/each. *: p<0.05, **: p<0.01, n.s.: not significant.

(C) The ratio of signal intensity of s-tdTomato in the nucleolus/cytosol (Nuc/Cyto). N=6~12 cells/each. *: p<0.05, **: p<0.01.

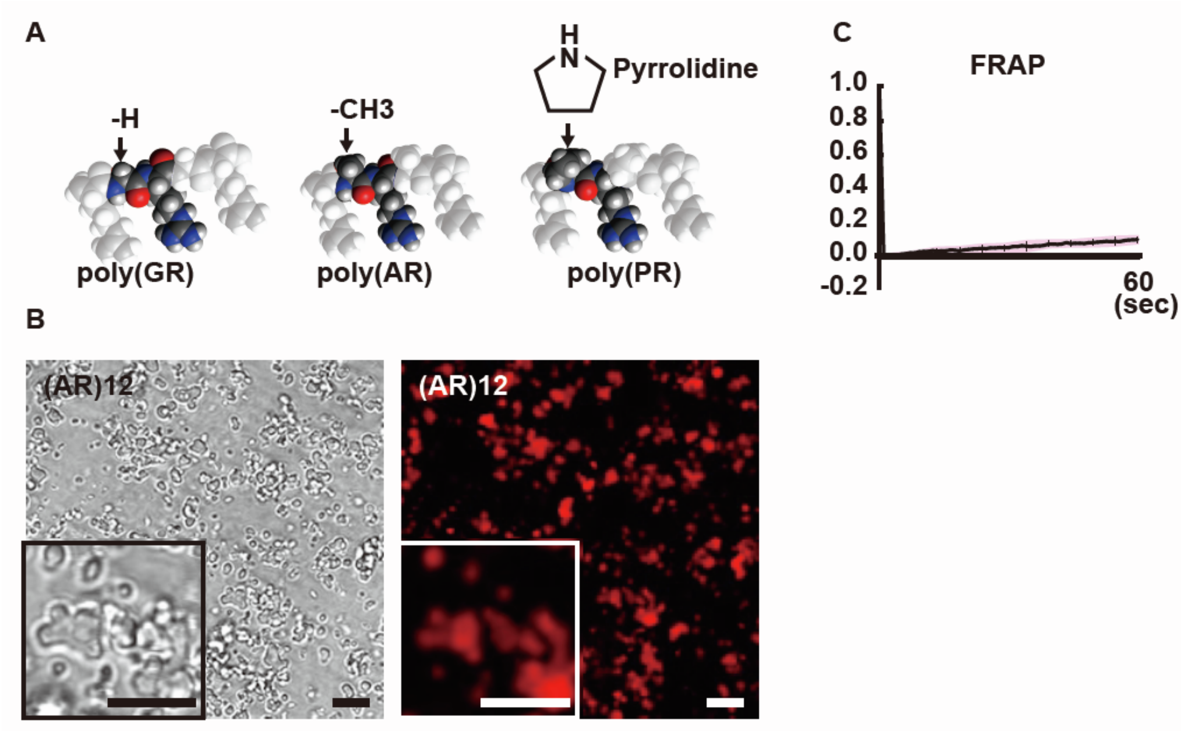

Figure S8. Biochemical properties of poly(AR)-mediated LLPS, related to Figure 5.

(A) Molecular structures of poly(GR), poly(AR) and poly(PR). Each dipeptide unit is shown in color. The side chain of each spacer amino acid is indicated.

(B) Representative images of LLPS droplets consisting of poly-rA and $(AR)_{12}$. Scale bar: 10 μm.

(C) FRAP recovery of TAMRA-RNA phase-separated with $(AR)_{12}$. Mean values and standard deviations from 6 independent analyses are shown.

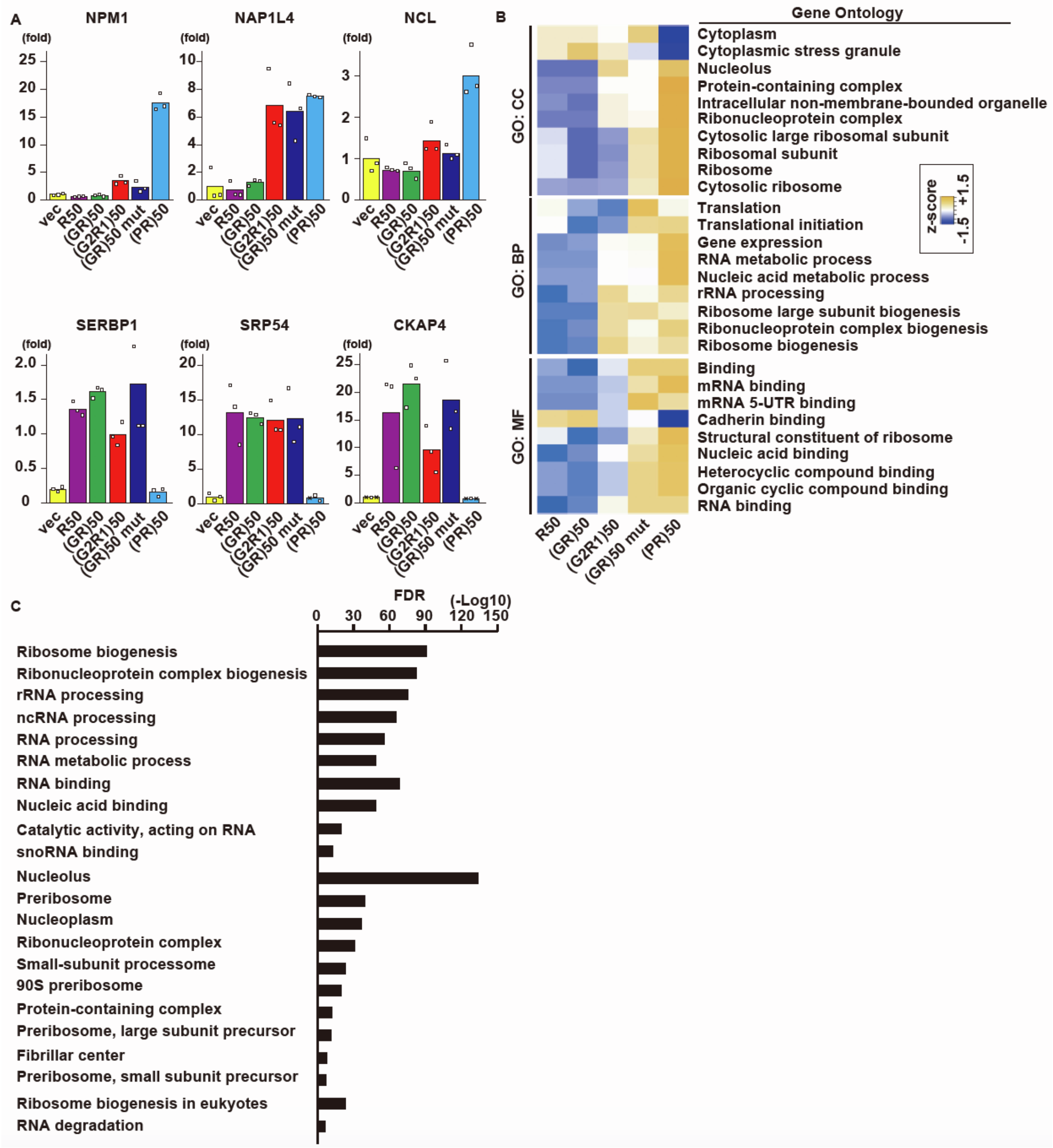

Figure S9. De-clustering of arginine charges contributes to differences in the interactomes of R-DPRs, related to Figure 6.

(A) Examples of signal intensities of LC/MS for identified proteins. Each bar shows the average of signal intensities from triplicate samples. White dots indicate the intensity of each sample and samples without signals are shown as “×”.

(B) Heatmaps indicating GO analyses. FDRs of each pathway were z-score-normalized and

heatmaps were drawn with Heatmapper [2].

(C) GO analysis of nucleolar proteins with signal intensities that were at least doubled in the interactome of $(PR)_{50}$ when compared with that of $(YR)_{50}$.

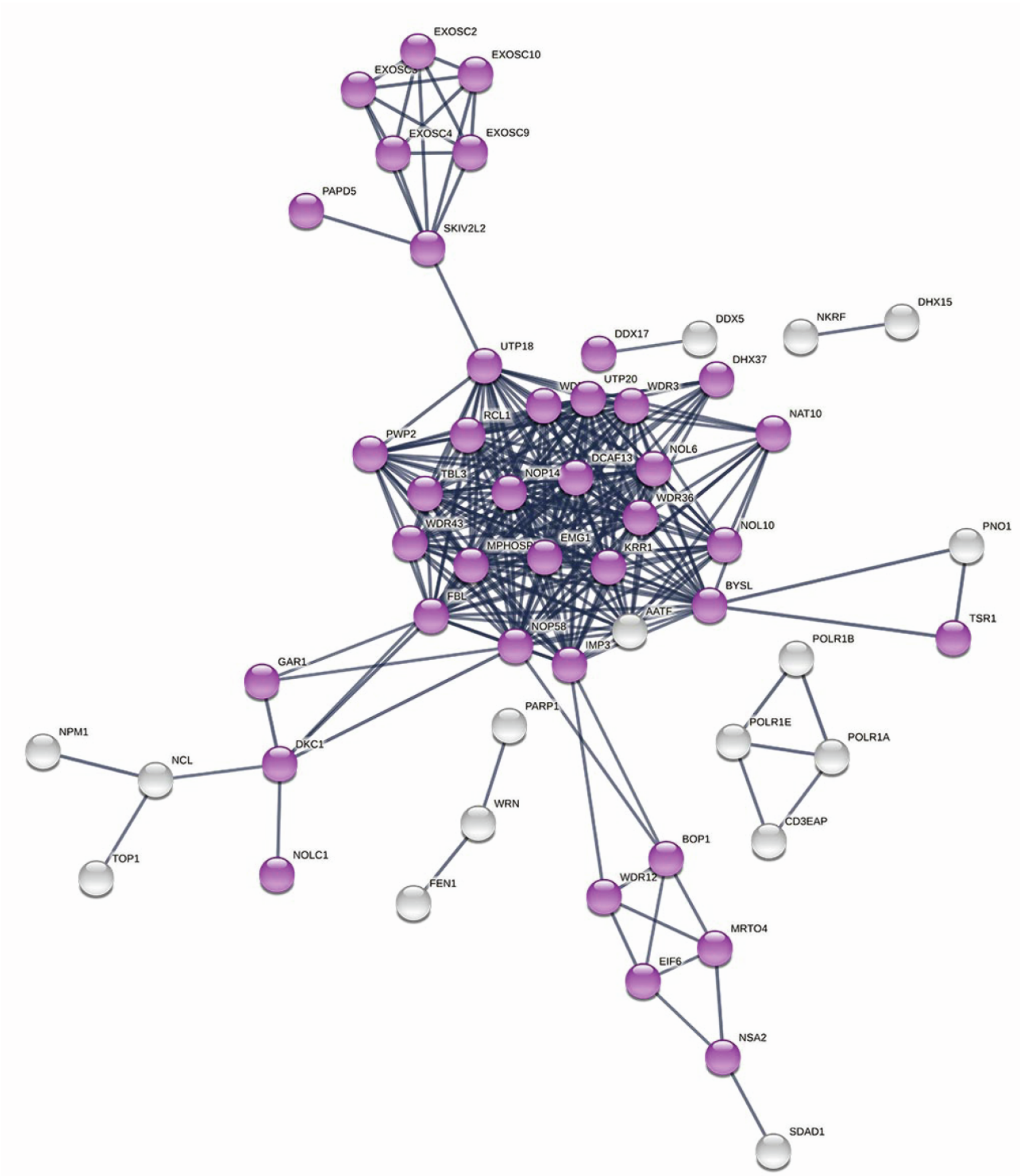

Figure S10. STRING interaction network of nucleolar proteins with signal intensities that were at least doubled in the interactome of $(PR)_{50}$, compared with that of $(YR)_{50}$, related to Figure 6.

Circles filled with magenta are proteins involved in rRNA processing (GO: 0006364, FDR=5.56×e-76).

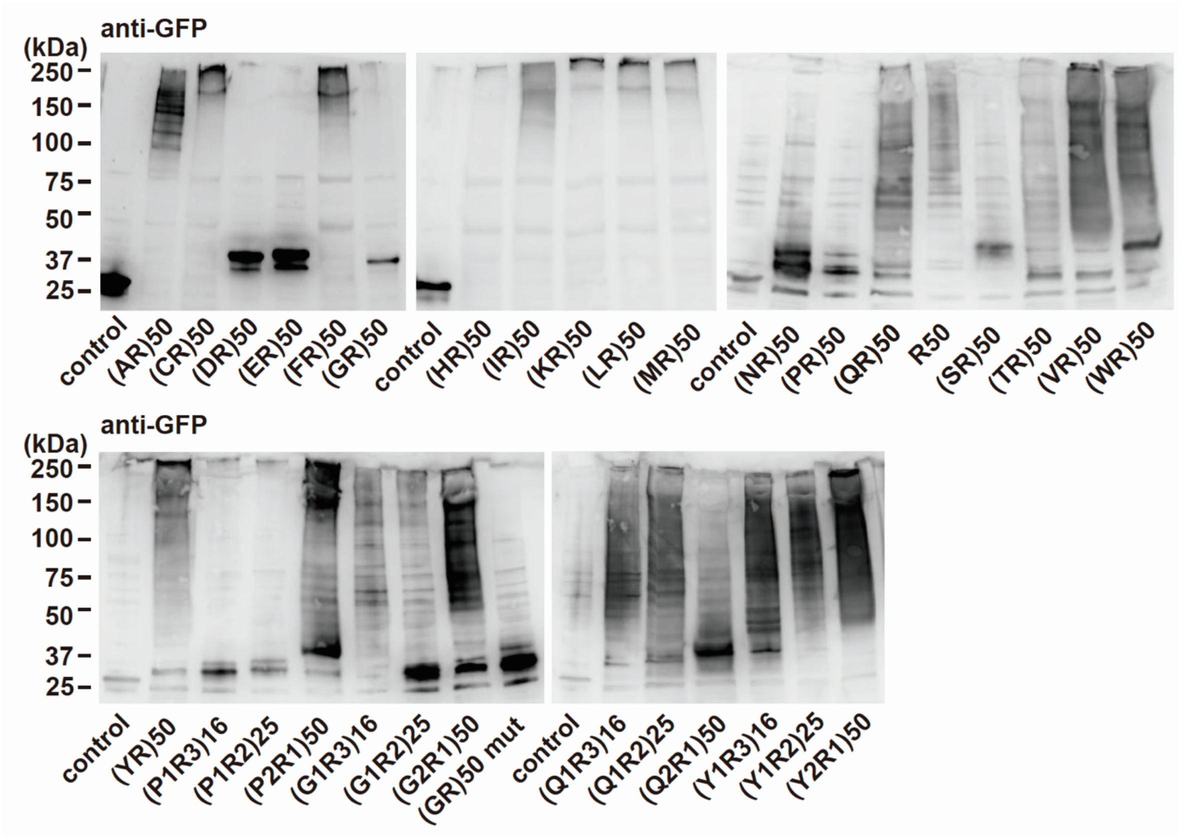

Figure S11. Immunoblot analyses confirmed expressions of all the $(XR)_{50}$ constructs, related to Figure 1, 2, 3, 5.

The lysate of the HEK293 cells expressing each GFP-(XR)50 construct was analyzed by immunoblot analysis using anti-GFP antibody. The lysate of the HEK293 cells expressing GFP vector was used as a control.

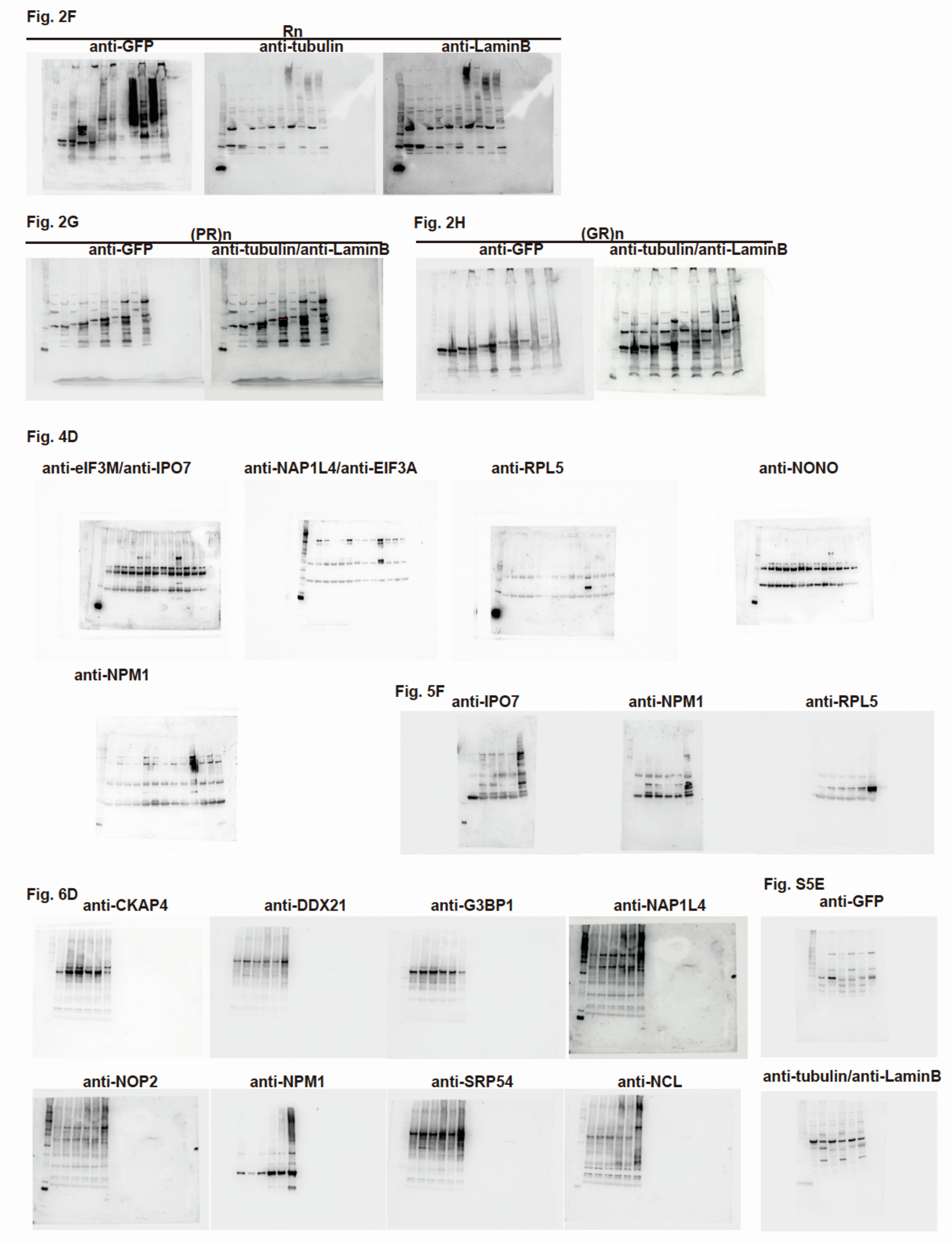

Figure S12. Uncropped images of immunoblot analyses demonstrated in this study, related to Figures. 2, 4, 5, 6, S5.

Supplemental references

1. Jumper, J., Evans, R., Pritzel, A., Green, T., Figurnov, M., Ronneberger, O., Tunyasuvunakool, K., Bates, R., Zidek, A., Potapenko, A., et al. (2021). Highly accurate protein structure prediction with AlphaFold. Nature *596*, 583–589. 10.1038/s41586-021-03819-2.
2. Babicki, S., Arndt, D., Marcu, A., Liang, Y., Grant, J.R., Maciejewski, A., and Wishart, D.S. (2016). Heatmapper: web-enabled heat mapping for all. Nucleic Acids Res. *44*, W147–153. 10.1093/nar/gkw419.

Table. S1. List of peptides.

Peptide		
KAPB2_H8	This study	N/A
$(PR)_{20}$	This study	N/A
$(GR)_{20}$	This study	N/A
$(GR)_{20}$ mut	This study	N/A
$(PR)_{12}$	This study	N/A
$(GR)_{12}$	This study	N/A
R_{12}	This study	N/A
FITC-$(PR)_{20}$	This study	N/A
FITC-$(GR)_{20}$	This study	N/A
FITC-$(GR)_{20}$ mut	This study	N/A
HA-tag peptide	MBL	3320-205
HA-R_{12}	This study	N/A
HA-$(AR)_{12}$	This study	N/A
HA-$(FR)_{12}$	This study	N/A
HA-$(GR)_{12}$	This study	N/A
HA-$(KR)_{12}$	This study	N/A
HA-$(LR)_{12}$	This study	N/A
HA-$(MR)_{12}$	This study	N/A
HA-$(NR)_{12}$	This study	N/A
HA-$(PR)_{12}$	This study	N/A
HA-$(QR)_{12}$	This study	N/A
HA-$(SR)_{12}$	This study	N/A
HA-$(YR)_{12}$	This study	N/A
